# Non-Invasive Glucose Sensing Technologies and Products: A Comprehensive Review for Researchers and Clinicians

**DOI:** 10.3390/s23229130

**Published:** 2023-11-12

**Authors:** Daria Di Filippo, Frédérique N. Sunstrum, Jawairia U. Khan, Alec W. Welsh

**Affiliations:** 1 Discipline of Women’s Health, School of Clinical Medicine, Faculty of Medicine, University of New South Wales, Sydney, NSW 2052, Australia; alec.welsh@unsw.edu.au; 2 Product Design, School of Design, Faculty of Design, Architecture and Built Environment, University of Technology Sydney, Sydney, NSW 2007, Australia; frederique.sunstrum@uts.edu.au; 3 Institute for Biomedical Materials and Devices, School of Mathematical and Physical Sciences, Faculty of Science, University of Technology Sydney, Sydney, NSW 2007, Australia; jawairia.khan@uts.edu.au; 4 Department of Maternal-Fetal Medicine, Royal Hospital for Women, Randwick, NSW 2031, Australia

**Keywords:** glucose sensing, non-invasive, continuous, intermittent, Diabetes Mellitus, product design and development

## Abstract

Diabetes Mellitus incidence and its negative outcomes have dramatically increased worldwide and are expected to further increase in the future due to a combination of environmental and social factors. Several methods of measuring glucose concentration in various body compartments have been described in the literature over the years. Continuous advances in technology open the road to novel measuring methods and innovative measurement sites. The aim of this comprehensive review is to report all the methods and products for non-invasive glucose measurement described in the literature over the past five years that have been tested on both human subjects/samples and tissue models. A literature review was performed in the MDPI database, with 243 articles reviewed and 124 included in a narrative summary. Different comparisons of techniques focused on the mechanism of action, measurement site, and machine learning application, outlining the main advantages and disadvantages described/expected so far. This review represents a comprehensive guide for clinicians and industrial designers to sum the most recent results in non-invasive glucose sensing techniques’ research and production to aid the progress in this promising field.

## 1. Introduction

Diabetes Mellitus (DM), defined by WHO as a chronic metabolic disease characterized by elevated levels of blood glucose, is increasing in incidence worldwide [1]. Currently ranked as the ninth cause of death, it affects 422 million people globally and 1.3 million people in Australia [2,3]. The number of people affected by DM is predicted to grow to 578 million people by 2030 and 700 million people by 2045 [4]. Yet, the diagnosis and management of Diabetes Mellitus is limited by low acceptability, compliance, and accuracy [5,6,7].

Currently, diabetes is diagnosed and monitored by different Invasive Glucose Sensing Technologies (IGST) depending on the type and age of screening. Type 1 DM, more common in childhood and early adulthood, is diagnosed with Random Blood Glucose (RBG) or HbA1c (which gives a picture of the glucose levels over the last 3 months) and can be confirmed with an Islet Cell Antibody (ICA-Ab) measurement (as against IA-2 (Insulinoma-Associated Protein 2), GAD65 (Glutamic Acid Decarboxylase 65), and ZnT8 (Zinc Transporter)). Its management includes Self Blood Glucose Monitoring (SBGM), Continuous Glucose Monitoring, and an insulin pump/pen for auto-injection to maintain blood glucose within the normal threshold.

Gestational DM, diagnosed with a 75 g Oral Glucose Tolerance Test (OGTT) (as per International Association of Diabetes and Pregnancy Study Group (IADPSG) guidelines) or with alternative tests for those women not tolerating the test, is managed by SBGM to check glucose levels and lifestyle changes (diet composition and physical activity) before considering the use of insulin and or metformin.

**Figure 1:**
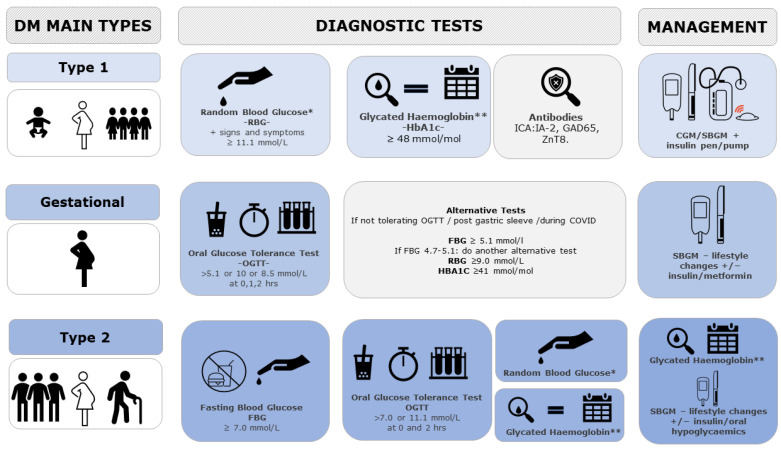
Diabetes Mellitus diagnosis and management. ICA—Islet Cell Autoantibodies, IA-2—Insulinoma-Associated Protein 2, GAD65—Glutamic Acid Decarboxylase 65, ZnT8—Zinc Transporter, CGM—Continuous Glucose Monitoring, SBGM—Self Blood Glucose Monitoring, FBG—Fasting Blood Glucose. Random Blood Glucose cut-off = 11.1 mmol/L, Glycated haemoglobin cutoff = 48 mmol/L.

Type 2 DM, more common in late adulthood, can be diagnosed with either Fasting Blood Glucose (FBG), OGTT (with different criteria for pregnant women), RBG, or Hba1c and monitored with SBGM/HbA1c [8]. For its management, lifestyle changes are trialed first before considering oral hypoglycemics (such as metformin and other classes) and insulin (see Figure 1). 

Apart from Continuous Glucose Monitoring (CGM), used for type 1 Diabetes Mellitus, none of the currently used techniques for Diabetes Mellitus diagnosis and management have optimal sensitivity/specificity and some have very poor acceptability [9]. The limited information provided by one-off measurements of blood glucose, either random, fasting, or after a set glucose load, have been repeatedly underlined in the literature [10]. Similar doubts have been described for the Self Blood Glucose Monitoring based on a few measurements per day [3,4,5,6,7,11].

The aim of this review is to comprehensively assess all the Non-Invasive Glucose Sensing Technologies (NIGST) reported in the literature (developed or in development), primarily from healthcare and industrial design perspectives, as well as describing the processes needed to produce new techniques. This review reports their mechanisms of action, their advantages and disadvantages, the evidence published regarding their clinical use, and the approaches used to research and design them. 

**Figure 2:**
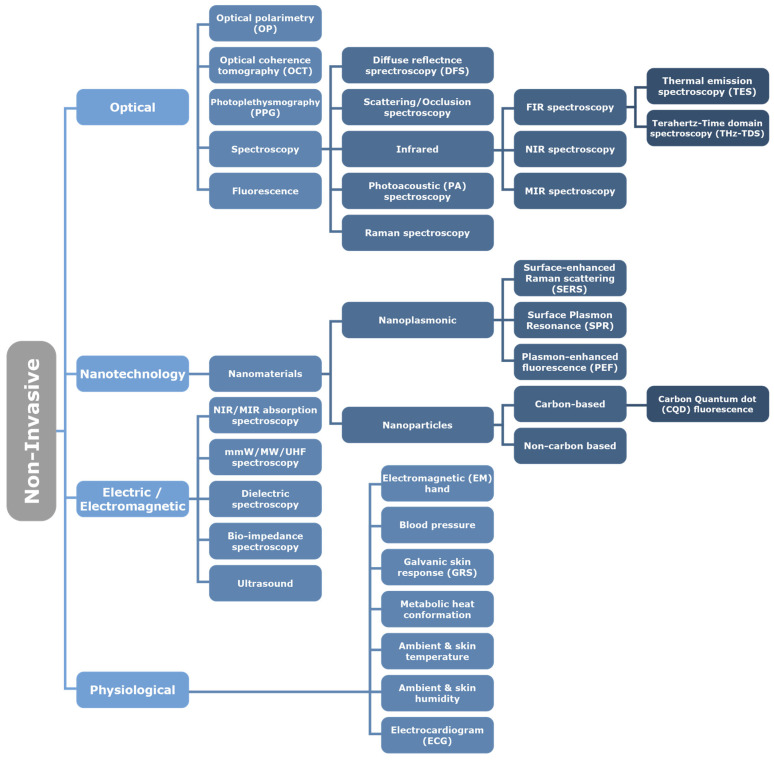
Classification of non-invasive glucose sensing technique.

Recent publications by Shang, Zhang [12], Hwang, Kang [13], Villena Gonzales, Mobashsher [14], and Chen, Zhao [15] divided glucose sensing techniques into invasive, minimally invasive, or non-invasive techniques. Minimally invasive techniques, using enzymes to detect glucose concentrations, are costly, unstable, and susceptible to factors such as humidity, temperature, and pH levels, and are not suitable for continuous measurement [16,17,18]. Our review focuses on NIGST, dividing them into optical, nanotechnology, electric/electromagnetic, and physiological techniques applied to glucose sensing (see Figure 2) and appraises all the commercial products under development, being trialed, and available on the market. 

This review extends the focus to the machine learning techniques applied to glucose sensing and highlights the potential of non-invasive glucose sensing techniques offering increased sensitivity and accuracy while minimizing the impact on users. Combining the expertise of three postdoctoral researchers in different fields (medicine, industrial design, and biomedical materials) has resulted in a comprehensive evaluation of NGIST in terms of acceptability, accuracy, and feasibility. Several new glucose sensing techniques offering good acceptability and sensitivity are emerging, among which Photoplethysmography (PPG) and Near Infrared (NIR) Spectroscopy have been identified as the most promising. Further research is recommended on developing, validating, and commercializing the most promising techniques.

## 2. Materials and Methods

To assess all the Non-Invasive Glucose Sensing Technologies (NIGST) reported in the literature (developed or in development) comprehending optical, nanotechnological, electric/electromagnetic, and physiological techniques, an online database search in MDPI (https://www.mdpi.com/) was run for the 5-year period between January 2018 and June 2023 with the keywords ““non-invasive” AND glucose monitoring OR glucose sensing” (55 (19 reviews, 36 articles)), “electromagnetic AND glucose monitoring OR “non-invasive” glucose sensing” (42 (13 reviews, 29 articles)), “optical AND glucose monitoring OR glucose sensing” (43 (14 reviews, 29 articles)), “nanostructure OR nanotechnology AND “non-invasive” AND glucose monitoring OR glucose sensing” (13) and “physiological AND glucose monitoring OR glucose sensing” (63). 

Next, a manual search of the references cited in these articles and the list of articles that have since cited these articles was conducted to identify additional relevant articles that may have been missed in the initial search. All identified articles were screened for relevance to non-invasive glucose monitoring before being included in the final analysis. The findings were categorized and summarized under the following headings: Optical (including Nanotechnology), Electric and Electromagnetic, and Physiological Techniques. Although Nanotechnology is considered in most reviews as a standalone category, there is some overlap with optical techniques. To avoid repetition, Surface-Enhanced Raman Spectroscopy, Surface Plasmon Resonance (SPR), Plasmon-Enhanced Fluorescence (PEF), and Carbon Quantum Dot (CQD) fluorescence are hence discussed in Section 4.1. 

NIGST techniques were reviewed from healthcare, industrial design, and biomaterial perspectives, analyzing their mechanism of action, their advantages and disadvantages, the evidence published regarding their clinical use, and the approach used to research and design them, also considering all the commercial products under development being trialed and available on the market for each technique. This review extends the focus to the machine learning techniques applied to glucose sensing and to the process needed to develop and commercialize new NIGST.

This process of citation tracking led to the identification of 25 additional articles. Removing the repeats and out-of-context articles, the total search yielded 124 journal articles. The breakdown of papers consisted of 37 articles for Electric and Electromagnetic, 54 articles for Optical, 29 articles for Nanotechnology, and 4 articles for Physiological that included the above keywords in the title and abstract.

## 3. Skin Layers and Properties

**Figure 3:**
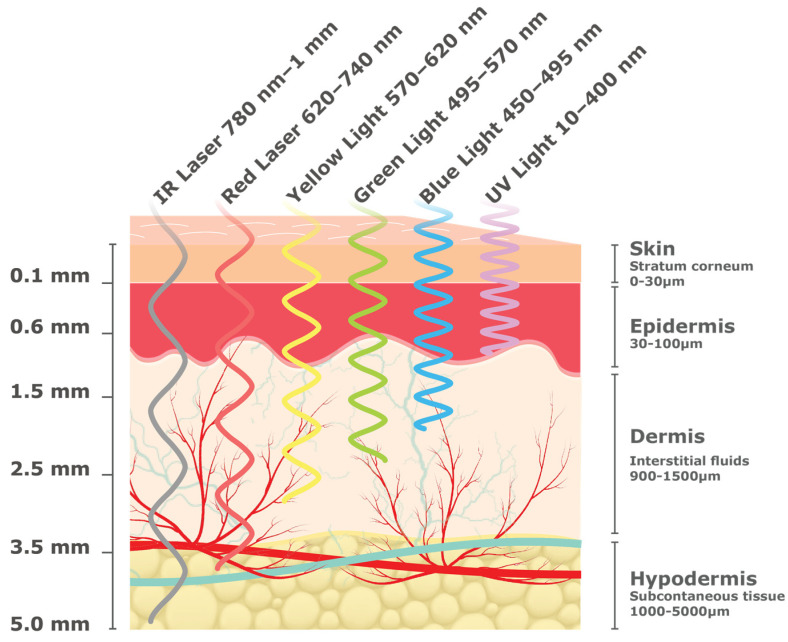
Schematic representation of the different skin layers and thicknesses.

The different NIGST incorporated in this review have unique interactions within the skin layers. The skin comprises seven layers and three distinct tissue types: the epidermis, dermis, and hypodermis, each with unique optical and electromagnetic properties that affect the penetration depth and the interaction with specific wavelengths and frequencies [19,20]. Such interactions can be analyzed to evaluate the tissue structure and properties, including the concentration of substances present in it, such as glucose, water, proteins, fats, electrolytes, etc. The different layers of skin thickness in micromillimeters are outlined in Figure 3. 

NIGST are based on different combinations of band, frequency, and wavelength to indirectly calculate the glucose concentration in the bloodstream. Table 1 illustrates the technique’s corresponding band, frequency, and wavelength. 

Electromagnetic and optical techniques both utilize wavelengths that are non-ionizing; however, each frequency range and wavelength offers distinct advantages and limitations within each technique. Within optical techniques, Terahertz (THz) can penetrate the skin superficially, primarily interacting with the outermost layer (the epidermis), and its penetration can be affected by light intensity/polarization, wavelength, and tissue properties [21]. While electromagnetic millimeter and Radio Frequency (RF) waves have lower frequencies, they offer high penetration depths to the subcutaneous tissue, providing valuable information on glucose distribution, although their accuracy can be impacted by the various substances encountered within the various skin layers.

When optical or electromagnetic waves travel through biological tissue, there are four primary interactions that can occur: reflection, scattering, absorption, and transmission [22]. In reflection-based methods, light or waves are directed onto the tissue surface and reflect off it. The intensity and characteristics of the reflected light or waves are analyzed. Scattering methods involve directing light or waves into the tissue, where it interacts with tissue structures, scatters, and emerges from different angles. Absorption methods use light or electromagnetic waves to probe tissue components that absorb specific wavelengths. The amount of absorbed light or waves provides information about the concentration of absorbers. Finally, transmission involves the passage of light or waves through tissue. The light or waves interact with various molecules, where some light or waves are absorbed by these molecules while the rest are transmitted through the tissue [20]. 

By analyzing the behavior of light or electromagnetic waves as they interact within the tissue, information can be gathered about the tissue and its components, including glucose concentration. Determining the interaction can depend on the specific wavelength used, the tissue’s properties, and the technique employed. Table 2 describes the four types of interactions (with light and glucose), the obtained information, and the techniques based on each with their challenges.

## 4. Techniques

### 4.1. Optical and Nanotechnology Techniques

With optical techniques, light interacts with different surfaces, particles, and materials in various ways and can be either reflected, scattered, or absorbed to directly or indirectly measure glucose levels. According to the law of reflection, when light is reflected, the light reflected is proportionate to the angle of the incident light. Scattered light can be explained by Rayleigh’s law, which describes how shorter wavelengths scatter more than longer wavelengths, meaning that scattered light is inversely proportionate to its wavelengths. Absorption and transmission is described by Beer–Lambert’s law, for which the amount of light absorbed by a sample is directly proportional to the concentration of the absorbing substance (in this case glucose) present in it [23]. 

Optical techniques have the potential for continuous and real-time glucose monitoring [18]. The optical techniques discussed include Optical Coherence Tomography (OCT), Optical Polarimetry, Photoplethysmography (PPG), and Spectroscopy (NIR, MID, FIR, NIR/MID, scattering/occlusion, photoacoustic, and diffuse reflectance) techniques. 

#### 4.1.1. Optical Coherence Tomography (OCT)

**Figure 4:**
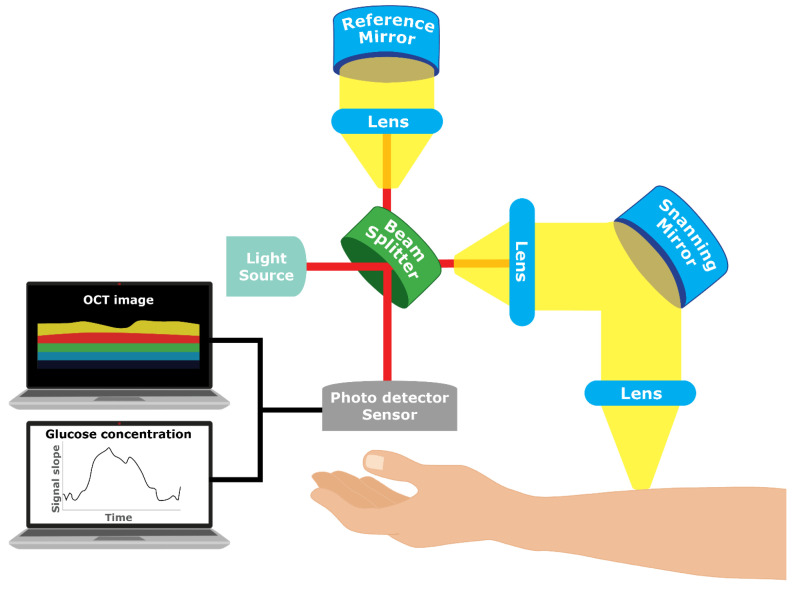
Optical Coherence Tomography (OCT) technique. The color codes in the OCT image (i.e., yellow, red, green, etc.) are attributed to the tissues and substances exhibiting different levels of reflectivity or scattering of light.

OCT has been historically used for ocular imaging and more recently applied to skin imaging for glucose detection [14,24,25]. OCT uses the interferometric phenomena (interference of light) to analyze how the light is backscattered and reflected by tissue (Figure 4). 

The Near-Infrared light reflected is measured and used to create an image of the tissue to reveal its internal structure considering the optical rotation angle of the glucose molecule [26]. The technique demonstrates good correlation between changes in the slope of OCT signals and blood glucose concentration at high resolution [18].

To analyze glucose levels through a fingertip device, Chen, Lo [27] used a differential Mueller model algorithm to analyze the rotation angle and depolarization index of the incident light. Miura, Seiyama [28] improved the accuracy of OCT with a low magnification objective lens of the OCT (LM-OCT) technique by reducing spatial resolution, making it a promising tool. Despite OCT’s advantages of good Signal-to-Noise Ratio (SNR), which is unaffected by heartrate, blood pressure, osmolytes, or red blood cell ratio, its disadvantages of low sensitivity, high sensitivity to movement, and skin temperature have limited progress in continuous monitoring devices [25,28]. Further research on the accuracy of OCT is needed to extract precise glucose levels.

#### 4.1.2. Optical Polarimetry (OP)

Glucose molecules have a specific ability to rotate the plane of light in a predictable way [29,30]. Optical Polarimetry (OP) measures the interaction of reflections and polarization of light and involves beaming polarized light at the aqueous humor within the eye to analyze the rotation and absorption energy of polarized light plane reflected from the glucose molecules with a photosensor detector, extracting information about the glucose concentration. 

**Figure 5:**
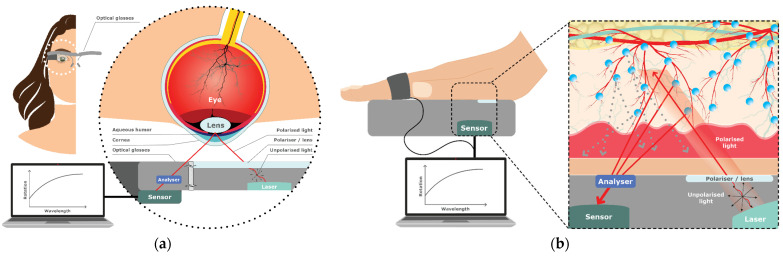
Optical Polarimetry (OP) sensing: (**a**) hypothetical continuous wearable optical lens; (**b**) intermittent prototype of a palm and finger sensor [31].

Figure 5a illustrates a hypothetical continuous wearable product example using OP. Hwang, Kang [13] developed a noncontact glucometer device that consisted of a light source, beam splitters, photodetectors, and a processing unit. The researchers directed a beam of polarized light at a wavelength of 1650 nm and power of 5 mW in the eyes of four rabbits. Measuring the rotation and absorption energy while analyzing serum glucose levels of the rabbits, the study found a high correlation between glucose level measurements, with mean differences of 8 mg/dL and 29.2 mg/dL for the in vitro and in vivo measurements. Although the aqueous humor demonstrated low protein and red blood cell count, using a photothermal detector to analyze glucose levels could potentially cause retinal damage. 

Li, Bai [31] developed a prototype of a novel, accurate, low-cost (<USD 250), and compact palm and finger sensor that analyzes weak polarized light optical rotation signals to estimate glucose concentrations using OP (Figure 5b). Using a generic Boosted Trees Regression learning model (see Section 4.4 on machine learning) with multiple light intensities and wavelengths to mitigate individual variability, the researchers demonstrated the plausibility of OP through skin tissue.

Advantages of optical polarimetry is that it is accurate, has high resolution, and can be relatively small in size [13,14,25]. The disadvantages are that it can be hard to obtain good optical rotation signals when the blood glucose levels are low. To counteract this, Wen, Lei [32] developed a fast and accurate spatial polarization modulation system (SPMS) that assesses a single digital image to identify low glucose levels. With the help of a rotating polarizer and an optical phase retarder, the weak optical rotation signals are made stronger and have shown that the SPMS has a resolution of 100 mg/dL. 

However, the presence of albumin concentrations in the interstitial fluid can skew accurate results. Stark, Arrieta [33] developed a broadband polarimeter setup that uses a Partial Least Squares (PLS) regression algorithm to distinguish between glucose and albumin based on their optical property characteristics. The method showed improved accuracy, allowing for more precise prediction of glucose concentration in the presence of albumin. Nevertheless, to improve optical polarimetry, further research is needed to increase the lag times and its high sensitivity to motion, temperature, interference with active compounds, and pH levels [25].

#### 4.1.3. Photoplethysmography (PPG)

**Figure 6:**
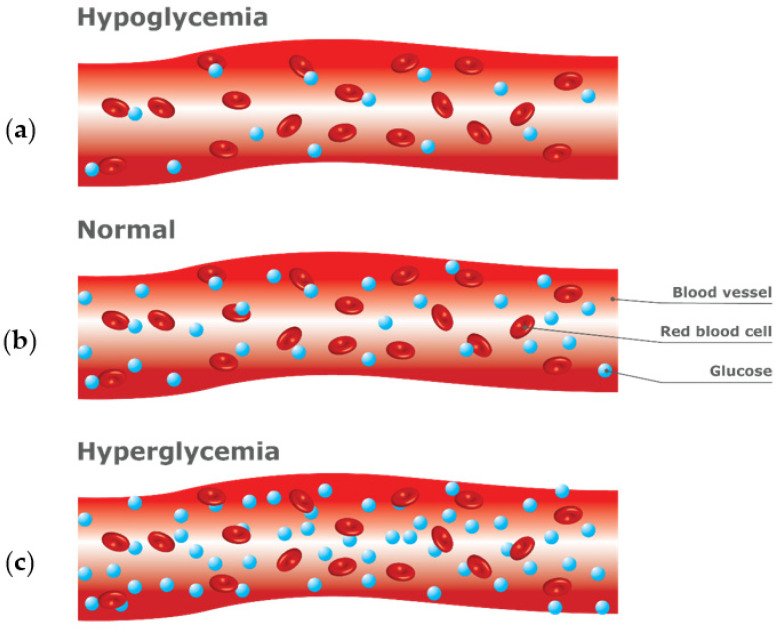
The presence of glucose within the circulatory system in individuals with (**a**) hypoglycemia, (**b**) normal glucose levels, and (**c**) hyperglycemia.

PPG is a low-cost technique that consists of an infrared emission of LED light onto the skin surface and measuring of the amount of light that is either transmitted or reflected back using a photodetector [34,35,36]. The amount of light absorbed or reflected by the tissue varies with changes in blood volume and flow, regulated by the autonomic nervous system and indirectly by changes in blood glucose levels [37]. When glucose levels rise, blood vessels dilate and blood flow increases, resulting in an increase in blood volume and an increase in light absorbed or reflected (Figure 6). Conversely, when glucose levels fall, blood vessels constrict and blood flow decreases, resulting in a decrease in blood volume and a decrease in light absorption or reflection. However, it is highly sensitive to motion or movement, hindering its accuracy of measurement [38]. Despite this limitation, PPG has been investigated as an NIGST for a continuous glucose monitoring device on the wrist or forearm by VitalSpex Pro & Bioptx Band, WristTee, LIFELEAF, and Glutrac (see Section 4.6 on product classification).

**Figure 7:**
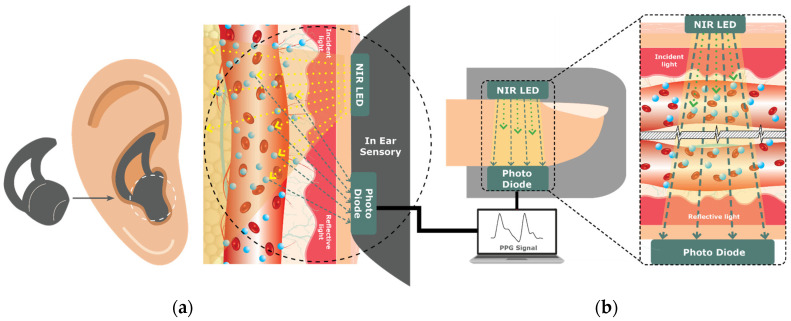
Photoplethysmography (PPG) technique illustrated as: (**a**) a continuous proof-of-concept ear device developed by Hammour and Mandic (2023); (**b**) an intermittent prototype of a finger sensor device NBM-200G (in development by OrSense, Raleigh, NC, USA).

More recently, proof-of-concepts measuring glucose levels using PPG have been studied through the ear canal (Figure 7a) and fingertip (Figure 7b) [35,36,39]. Hammour and Mandic [39] developed a proof-of-concept in-ear device equipped with a low-cost pulse oximeter that enables continuous and non-invasive blood glucose measurement (Sanmina by Sanmina Corp., San Jose, CA, USA)).The device utilizes an infrared LED and a PPG chip (connected to a data acquisition board with Bluetooth capability) to collect and process real-time data for blood glucose estimation. While capturing glucose levels with a commercially available glucometer, the proof-of-concept device achieved 82% accuracy within the clinically acceptable range. Due to its stable temperature, constant pressure, consistent positioning, and universal fit, the ear canal makes it a promising, cost-effective, and scalable solution for continuous and non-invasive blood glucose monitoring [39].

Using a smartphone video-based technique, Islam, Ahmed [36] converted videos of the fingertip into PPG signals while removing noise and interference (i.e., peaks, time differences, and derivatives) using different regression models for improved accuracy of glucose level prediction. Among the models, the Partial Least Square Regression (PLS) (see Section 4.4 on machine learning) showed the lowest Standard Error of Prediction (SEP) at 17.02 mg/dL when tested on an unbiased dataset.

PPG absorbance and reflectance signal can vary depending on blood circulation, blood viscosity, peripheral vascular resistance, vascular elasticity, and movement [37]. To increase the accuracy of this technique, machine learning or other paired sensor techniques would be needed to adjust for these variables [40]. 

Yen, Chen [41] found that combining dual-wavelength PPG with another method, such as Bioelectrical Impedance (BI), delivered quantitative results. Although BI does not directly measure glucose levels, it can provide information about body composition and fluid distribution (i.e., body fat, mass, and water). It was suggested that further testing would be needed; however, the proposed method performed better than only using PPG to detect glucose levels. Additionally, Haque, Hossain [35] optimized a fingertip model design and accuracy by using a Monte Carlo photon simulation-based model and machine-learning model (XGBoost) (see Section 4.4 on machine learning) for estimating a blood-glucose concentration model, which showed better performance compared with previous models. Wei, Ling [42] described the analysis of Photoplethysmography (PPG) data from a wearable device based on a technique called joint averaging in the feature domain and piecewise feature selection method to improve the accuracy of the computer program. To remove any noise or interference from the measurements, a bit plane Singular Spectrum Analysis (SSA) was implemented. This assisted in eliminating errors caused by environmental factors and found that it was more accurate than a simple Random Forest regression model. They also found that 87.06% of the data fell on zone A of the Clarke Error Grid, and there was a Mean Absolute Relative Difference (MARD) of 12.19% (see Section 4.6 on product classification for detailed explanation of accuracy assessment).

#### 4.1.4. Spectroscopy

Spectroscopy is a technique that measures the interaction with light (absorption and transmittance) [26]. By measuring the absorption, reflection, or scattering of light at specific wavelengths, the concentration of glucose in the body can be determined without the need for invasive methods. Spectroscopy is suitable for glucose monitoring as it enables real-time and non-invasive measurement of glucose levels, providing valuable information for diabetes detection and management. 

The techniques discussed include Near-Infrared Spectroscopy (NIRS), Mid-Infrared Spectroscopy (MIRS), Far-Infrared Spectroscopy (FARS; including Thermal Emission Spectroscopy and Terahertz-Time Domain Spectroscopy [THz-TDS]), Scattering/Occlusion Spectroscopy, Diffuse Reflectance Spectroscopy (DRS), Photoacoustic Spectroscopy (PAS), and Raman Spectroscopy.

#### 4.1.5. Near-Infrared (NIR) Spectroscopy

Considered one of the most effective techniques, NIR Spectroscopy shines light in the range of 750 nm to 2.5 µm onto the skin, such as the fingerprint, and the reflected light is measured [43,44,45,46,47], with accurate in vivo measurements demonstrated at 1550 nm for this particular technique [48]. Glucose molecules can absorb light, resulting in changes in the intensity of the reflected light at specific wavelengths, which are used to estimate the glucose concentration. Because of its low cost, compact nature, and high penetration level, it is a highly promising technique and has been the technique of choice for numerous devices, such as Wizmi^TM^ by Wear2b Ltd. (Rosh Pina, Israel), Glucose Tracker Clip by AnnNIGM (Moscow, Russia), Brolis Sensor Technology by BROLIS (Vilnius, Lithuania), GluControl GC300 by ArithMed GmbH and Samsung Fine Chemicals Co., Ltd. (Seoul, Republic of Korea), HELO Extense by WOR(I)D World Global Network Corp. (Miami, FL, USA), GlucoStation by GlucoStation (Wroclaw, Poland), Sensys GTS by Sensys Medical Inc. (Chandler, AZ, USA), TouchTrackPro (Chandler, AZ, USA), and DioMonTech’s range of devices, D-Sensor, D-Pocket, and D-Base (Berlin, Germany) [19,26,49] (see Section 4.6 on product classification). 

**Figure 8:**
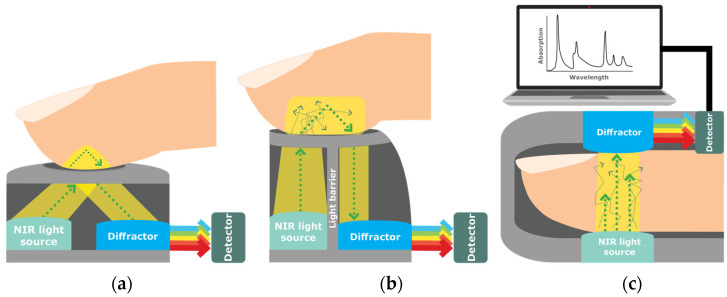
Near Infrared (NIR) Spectroscopy illustrated through three instruments of measurement through the finger: (**a**) reflectance; (**b**) interactance; and (**c**) transmittance. The spectrum of colors is attributed to when light passes through the diffractor, dispersion occurs causing different colors to separate due to varying wavelengths, which is analyzed by the detector.

NIR uses the dispersive spectrometer and can be measured by reflectance (reflecting light on the tissue at an angle; Figure 8a), interactance (a compromise between the two, where light is separated by a seal; Figure 8b), and transmittance (going through the tissue from one end to the other, such as through the finger; Figure 8c) [14,45]. A current research focus is on reflectance spectra for glucose measurement of the inner lip using NIR Spectroscopy [43]. To predict glucose concentration, Heise, Delbeck [43] employed a calibration method, which provides direct measurements of glucose concentration in the tissue independent of dynamic glucose transport processes. Further research on NIR is needed to account for the diffusion processes of different substances within the tissue that may impact glucose levels’ measurement. Another limitation of NIR impacting accuracy and reproducibility is its sensitivity to physiological parameters and environment factors, such as tissue thickness, temperature (skin and ambient), skin (tone and melanin), substances in the tissue (fat, protein, and water), and ambient light intensity [14,50,51]. 

To accurately measure glucose levels, the technique requires a multivariant analysis to adequately detect these varying attributes [51]. In one study, higher accuracy was found when using NIR Spectroscopy alongside SpO_2_ and heartrate in a compact fingertip sensor [49]. In addition, skin and tissue variations have been investigated to increase the accuracy of measurements [19,23]. Using the Monte Carlo simulation method (see Section 4.4 on machine learning), one study employed a dual-channel measurement for glucose and noise signal for different skin colors [19]. The study investigated different melanin concentrations in the skin, considering optical properties such as absorption coefficient, scattering coefficient, and refractive index. The study determined that longer wavelengths with longer source-detector separations yielded better signal-to-noise ratios in darker skin, while optimal wavelength and separations varied for light and medium skin, requiring shorter separations and specific wavelengths for the short and long channels. Further research is needed to verify this in vivo. 

#### 4.1.6. Mid-Infrared (MIR) Spectroscopy

Like NIR Spectroscopy, MIR works on the principle that different molecules, including glucose, absorb/transmit light at specific wavelengths, resulting in unique spectral fingerprints. However, unlike NIR, MIR is a high-cost technique [26]. MIR involves emitting the narrowband light source Quantum Cascade Laser (QCL) onto the skin surface, causing glucose molecules in the interstitial fluid to vibrate, and measuring the amount of light that is either transmitted or reflected. MIR Spectroscopy uses light in the range of 2.5 to 25 µm and penetration depths can go up to 100 μm without causing irritation or discomfort [44,45,52]. The absorption spectra of glucose in this range are more distinct and easily distinguishable from other compounds in the skin, which makes it an ideal method for glucose monitoring. 

Combining MID with Photoacoustic Spectroscopy has great potential for non-invasive glucose monitoring [53]. Specifically, Lubinski, Plotka [52] are currently working towards the development of a handheld device, D-Base by DioMonTech AG(Berlin, Germany), for diabetic patients. The novel device combines Photothermal and Photoacoustic Spectroscopy with low-power MIR Spectroscopy in wavelength depths of 8 to 11 μm directing onto the skin, such as the finger, thumb, hand, or wrist, to detect the dissipated heat energy produced by the vibrated glucose molecule. The heat energy produced is detected by a photoacoustic and photothermal deflection sensor and analyzed by an algorithm and machine learning model (XGBoost algorithm) to determine glucose levels. The results from 100 diabetic and healthy volunteers showed that the non-invasive method was very accurate, with less than 1% of the data falling outside of the highest accuracy zones (see Section 4.6 on product classification). 

#### 4.1.7. NIR/MID Absorption Spectroscopy

**Figure 9:**
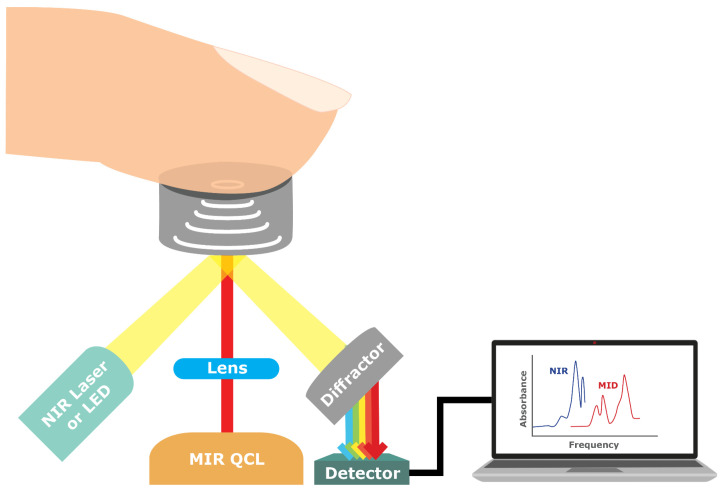
Near Infrared/Mid Infrared (NIR/MID) Absorbance Spectroscopy.

NIR/MID Absorption Spectroscopy combines and uses aspects from both Near-Infrared (NIR) and Mid-Infrared (MID) light to capture a more comprehensive spectrum of absorption by glucose molecules in body tissues, including the most abundant substance, water [54]. By analyzing the amount of light absorbed at these specific wavelengths, the concentration of glucose in the tissues can be determined (Figure 9).

Shokrekhodaei, Cistola [29] employed a multiwavelength measurement in the Visible (VIS) and NIR range with machine learning to increase sensitivity and selectivity using four wavelengths (485, 645, 860, and 940 nm) that correlated with the predictability and accuracy of in vitro sensing of glucose concentrations in water (albumin PBS solutions). Mandal and Manasreh [55] found a higher accuracy with two peak wavelengths of 535 nm and 593 nm based on molar absorption coefficients alongside a photodiode (to measure the transmitted intensity) and a microcontroller to estimate the HbA1c percentage based on changes in absorbance at different wavelengths. 

#### 4.1.8. Far-Infrared (FIR) Spectroscopy, Terahertz-Time Domain Spectroscopy (Thz-TDS), Time of Flight (TOF), and Thermal Emission Spectroscopy (TES)

Also known as Terahertz Spectroscopy, FIR uses light between Far-Infrared (FIR) and Microwaves (MW) in the range of 0.3 to 30 THz that penetrate from 25 to 1000 µm to analyze how glucose molecules absorb specific frequencies of FIR radiation, which corresponds to their vibrational and rotational transitions [26,46]. The technique is susceptible to strong light absorbance from water, thus skewing accurate glucose results. In comparison, Terahertz-Time Domain Spectroscopy (Thz-TDS) has excellent water sensitivity and non-ionizing photon energy [56]. 

**Figure 10:**
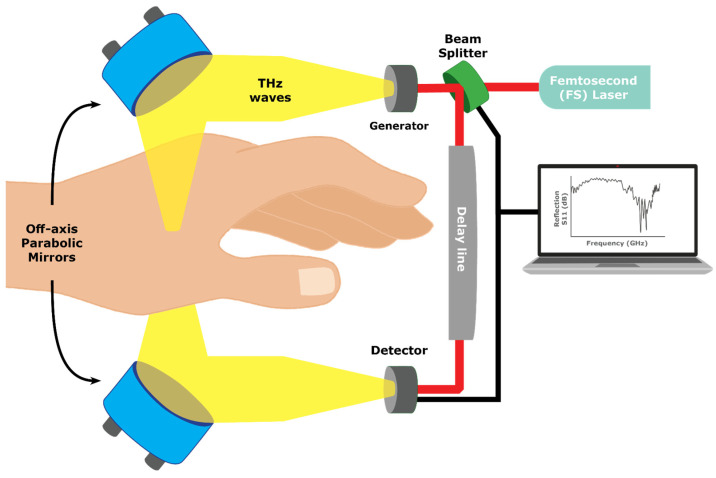
Terahertz-Time Domain Spectroscopy (Thz-TDS).

Thz-TDS and Time Of Flight (TOF) are techniques that use short pulses from a Femtosecond (FS) laser directed into the tissue and measure the time delay and magnitude of the reflected or transmitted light as it interacts with glucose molecules in the body [26]. The magnitude of the delay and the proportion of energy absorbed by the glucose molecules are measured to analyze the concentration of glucose in the body (Figure 10). 

The difference between TOF and Thz-TDS is that THz-TDS has an additional capability to sweep through a wide range of frequencies, allowing it to gather more detailed information about the material’s properties [14]. THz-TDS can measure the refractive index and the spectrum of material interaction with light at different frequencies. Kaurav, Koul [57] developed a sub-THz glucose sensor using a waveguide probe sensor unit to analyze glucose levels of a phantom model. While analyzing time-domain responses using a Levenberg–Marquardt algorithmic Back Propagation Neural Network (BPNN), the glucose sensor provided a low-cost, accurate, and highly sensitive to 2 decibels (dB) glucose measurement system compared with Infrared, Microwave (MW), or Millimeter Wave (mmW) sensors found in the literature, and has the potential to be implemented as an earlobe device. 

However, there is a significant obstacle in developing Terahertz (THz) sensors that can detect analytes with high sensitivity due to the substantial difference between the THz wavelength and analyte thickness or quantity within the tissue [58]. Al-Naib [58] addressed this obstacle by developing a Terahertz meta-surface based on s-shaped complementary resonators to investigate glucose concentration levels associated with different diabetes conditions. The research showed significant resonance frequency redshifts of 110.6 GHz and improved wavelength sensitivity compared with previous studies, making it a potential tool for detecting hypoglycemia and hyperglycemia.

**Figure 11:**
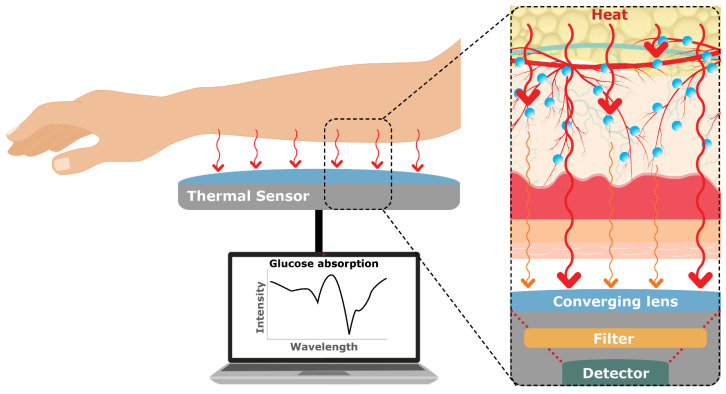
Thermal Emission Spectroscopy (TES).

Finally, Thermal Emission Spectroscopy (TES) involves measuring the infrared radiation emitted by the body (8–14 µm) to analyze the glucose levels at 9.4 µm [59]. TES relies on the principle that glucose levels affect the body’s heat distribution, which in turn affects the emitted thermal radiation (Figure 11). 

Therefore, no penetration of light or wavelengths is needed for this measurement technique and it does not require calibration [60]. To explain further, glucose molecules have specific absorption characteristics in the infrared region, and, by detecting and analyzing the emitted thermal radiation, it is possible to indirectly estimate glucose concentrations. By studying the spectral properties of the emitted infrared radiation, TES can provide information about glucose variations in a non-invasive manner. However, its sensitivity to temperature and movement, measurement variability when it comes to tissue thickness, and real-time capability need further investigation to be suitable for non-invasive glucose monitoring [14].

#### 4.1.9. Scattering/Occlusion Spectroscopy

Scattering/Occlusion Spectroscopy measures the interaction (scattering) of light waves through matter. In this technique, the red or NIR light interacts with a sample, and the resulting signal is analyzed to obtain information about the sample’s composition [30]. For Scattering Spectroscopy, the higher the glucose levels in the blood, the more scattering, and vice versa. 

**Figure 12:**
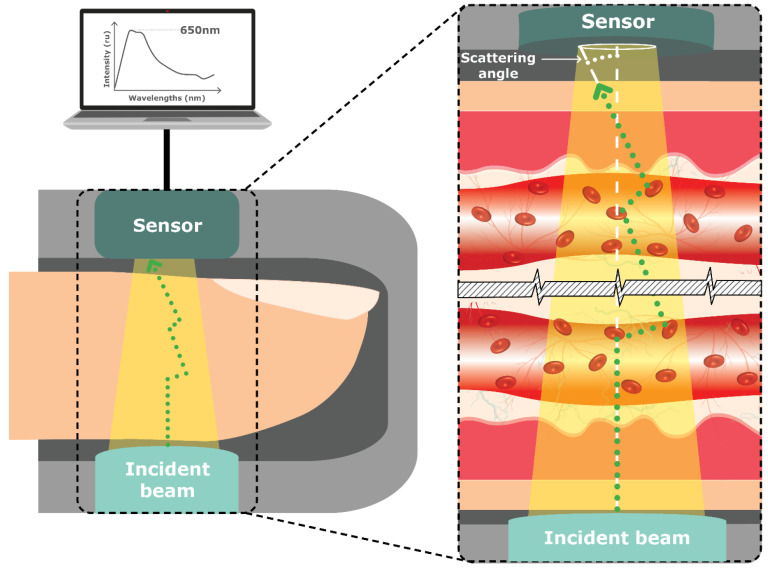
A schematic representation of a finger sensing device using Occlusion Spectroscopy.

On the other hand, Occlusion Spectroscopy applies pressure on the tissue of measurement. For example, the device NBM 200 by OrSense Ltd. (Raleigh, NC, USA) uses a probe and finger placement, such as the thumb, through a device where Red/NIR light sources, detectors, and pneumatic cuffs temporary restrict (occlude) blood flow and generate a dynamic optical signal for accurate monitoring [61,62]. The method leverages transmission modes, dynamic signal generations, multispectral data, and sophisticated algorithms to enhance sensitivity, specificity, and accuracy in glucose measurement (see Figure 12). 

The advantage of Occlusion/Scattering Spectroscopy is that it is highly sensitive, safe, convenient, allows for real-time monitoring, and has minimal interference. Conversely, variations in tissue protein, fat, red blood cell aggregation (erythrocytes), blood osmolality, skin between sex, age, and oxygen saturation all affect glucose measurements and need further testing. These variations can be counteracted with the use of multiwavelength analysis, sensing the variations as well as a multivariant analysis machine learning algorithm enabling to filter the variations related to these features [30]. 

#### 4.1.10. Photoacoustic Spectroscopy (PAS)

**Figure 13:**
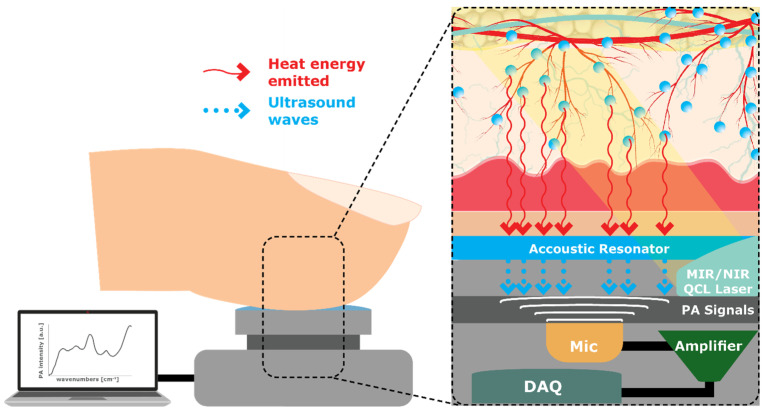
Photoacoustic Spectroscopy (PAS) finger sensor illustrating heat waves emitted from glucose molecules.

PAS uses light to measure the amount of glucose by shining an NIR and/or MIR laser beam on the skin (Figure 13). Glucose in the tissue, as well as other substances, absorbs the light emitting heat energy, creating a sound (acoustic) wave that can be detected by a microphone, which is then analyzed to determine glucose concentration [30]. PAS has a rapid response time, high sensitivity and selectivity, and is very precise [63]. One disadvantage of PAS is that other substances within the tissue, including water, fat, and proteins (albumin and hemoglobin), can increase light permeability (absorption), causing the sensitivity to be much higher, thus skewing the results of glucose concentration [64]. 

The region of the body for measurement needs careful consideration, as sensitivity increases with other substances present in the tissue. To enhance the detection sensitivity, Aloraynan, Rassel [65] developed a single-wavelength MIR QCL light source with frequency modulation wavelengths of 10 to 30 kHz, and scanned phantoms to generate acoustic waves that were detected and analyzed with machine learning classification. The experimental results showed the feasibility of the system for non-invasive glucose detection, serving as an initial step before further development for in vivo measurements. 

Another disadvantage of PAS is the weak acoustic signal detection of thermal waves. By optimizing a T-type opened resonator cell with frequencies of approximately 25 to 52 kHz, the detection of Photoacoustic (PA) signals can also be enhanced [63]. Although further testing is needed, with this enhancement, Tang, Ni [63] demonstrated higher resonance peaks, increased PA signal gain, and improved detection sensitivity compared with conventional cells, where this optimization method could guide the development of PAS by maximizing signal gain for non-invasive glucose monitoring.

#### 4.1.11. Diffuse Reflectance Spectroscopy (DRS)

**Figure 14:**
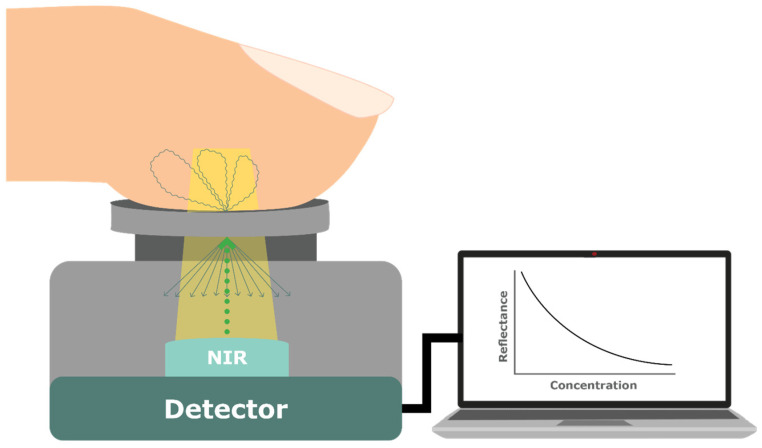
Diffuse Reflectance Spectroscopy technique illustrated in an intermittent finger sensor.

In DRS, light is directed onto the tissue surface, and the reflected light is measured to analyze the absorbance coefficient parameters of the underlying tissue (Figure 14) [66,67]. The diffuse reflection technique is preferred for evaluating skin samples in cases where spectral ranges encompassing data-rich attributes, such as first overtone and combination band vibrations, are being examined [43]. Using NIR wavelengths of 900–1300 nm on phantom tissue models, Hepriyadi and Nasution [66] found accurate readings of glucose concentration. As glucose has specific absorption characteristics in the near-infrared region, blood glucose levels change as a result, affecting the scattering and absorption of light in the tissue. 

DRS offers several advantages, such as real-time monitoring capability, potential for continuous glucose monitoring, and applicability to different tissue thicknesses [67], and is a promising technique currently undergoing testing in a wrist watch device by BioMKR (Prediktor Medical, Fredrikstad, Norway) and an early-stage continuous multisensory armband by Biovotion AG (Zürich, Switzerland; see Section 4.6 on product classification) [68]. Furthermore, Pozhar, Mikhailov [67] were able to measure small changes in glucose levels with an NIR DRS prototype (with a laser with two detector photodiodes), demonstrating DRS’s potential for non-invasive glucose sensing. DRS poses challenges due to interference from other tissue components and temperature drifts causing a delay and instability. Further research is needed on accurate calibration models to enhance the sensitivity and accuracy of this technique.

#### 4.1.12. Fluorescence

When excited by light, fluorophores emit fluorescence in proportion to the concentration of the glucose molecules bound to it [46]. This emitted fluorescence is then measured and analyzed and may represent a promising technique to determine the glucose concentration [17]. However, fluorescence is limited by photobleaching, where the fluorophore loses its ability to fluoresce over time due to continuous exposure to excitation light, and (currently) low accuracy [69]. Nonetheless, with further research, fluorescence has the potential to be a cost-effective non-invasive alternative to glucose monitoring. As discussed in the next paragraphs, fluorescence has been trialed within Nanotechnology for the development of NIGST. 

#### 4.1.13. Plasmon-Enhanced Fluorescence (PEF)

PEF combines the principles of Fluorescence Spectroscopy with Nanotechnology (nanoplasmonics) to enhance the sensitivity and accuracy of glucose detection. Nanotechnology holds the promise of revolutionizing non-invasive glucose monitoring techniques, offering a new frontier of possibilities in diabetes management [70]. One of the key advantages of Nanotechnology lies in its capacity to create ultra-small and precise devices, with dimensions less than 100 nm, capable of interacting with glucose molecules. This breakthrough enables the development of significantly smaller glucose sensors compared with conventional ones, enhancing their ability to detect even minor changes in glucose levels with high accuracy through the use of nanomaterials [71,72]. 

Nanomaterials have gained significant attention due to their unique properties and potential applications, as these materials offer several advantages for glucose sensing, exhibiting unique optical, electrical, or electrochemical properties that can enhance sensitivity, selectivity, and compatibility with wearable devices. Although research in nanomaterials has progressed in enzymatic techniques (classified in this paper as minimally invasive [12,13,14]), Plasmon-Enhanced Fluorescence (PEF) is categorized as an optical nanoplasmonics technique for glucose sensing.

Nanoplasmonics involves the oscillating field in the optical phenomena that studies the interaction of light with free electrons (plasmons) in nanoscale metallic structures [73,74]. Nanoplasmonics are highly sensitive, detecting glucose at low concentrations, and can be miniaturized for handheld/wearable devices. 

The principle behind Plasmon-Enhanced Fluorescence (PEF) for glucose monitoring involves functionalizing the surface of gold or silver plasmonic nanoparticles with glucose-specific receptors, such as a nanoantenna, that enhance the fluorescence signal emitted by fluorescent probes or molecules that are sensitive to glucose concentration [73]. It involves the interaction between plasmonic nanoparticles and fluorophores’ electromagnetic fields, resulting in increased fluorescence intensity. This technique offers the potential for real-time and label-free glucose monitoring; however, further research is needed on the technique. 

#### 4.1.14. Carbon Quantum Dot (CQD) Fluorescence

Carbon-based nanomaterials, such as Carbon Nanotubes (CNTs), Graphene Oxide (GO), and Carbon Quantum Dots (CNDs), have a growing interest in the literature due to their potential catalytic properties and non-toxic application compared with alternative metal-, oxide-, or other-based nanomaterials [75,76]. In particular, NIR-emitting CQDs, also known as Carbon Nanodots (CNDs), have superior properties, such as high resistance to photobleaching, excellent frequency/water dispersion and surface charge, and distinct chemical, fluorescent, and electronic properties [77]. 

CQDs rely on the fusion of carbon nanoparticles made of semiconducting nanomaterials, known as Quantum Dots (QDs) [78,79]. Using a combination of hydrothermal and microwave methods with a size of less than 10 nm that can be easily modified, CQDs are proving further to be cost effective, mobile, and versatile, while also displaying high stability, eco-friendliness, and biocompatibility [79,80,81,82,83]. 

**Figure 15:**
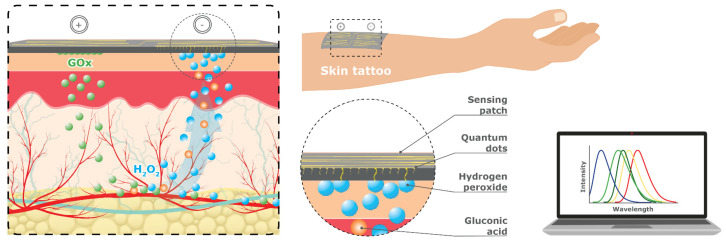
Carbon Quantum Dot (CQD) fluorescence tattoo-like sensor patch. CQDs exhibit fluorescence intensity changes in response to different glucose concentrations, allowing for sensitivity detection through spectral shifts and intensity alterations in emitted light.

CQDs can be deposited on nanosheets and a variety of different substrates, including nanoparticles, nanowires, and thin nanostructured films, to deliver a disposable patch for glucose sensing (Figure 15). Nanosheets are extremely thin two-dimensional materials made up of layers that are only a few atoms thick and are used as a sensing platform that can be attached to the skin like a temporary tattoo (i.e., sensing patch). Recent advancements in the fabrication and biomimetic surface of nanostructures [84] have yielded promising results, propelling the future of wearable patch devices. These cutting-edge technologies hold the potential to provide diabetic patients with more comfortable and convenient monitoring solutions while delivering superior accuracy, sensitivity, and reliability for glucose sensing. 

Cho and Park [85] used an excitation wavelength of 360 nm to examine changes in fluorescence emission from blue to green CDs that corresponded to glucose concentration. More recently, Li, Luo [86] developed a novel hydrogel optical fiber fluorescence sensor with segmental functionalization QDs, which allowed for simultaneous continuous monitoring of pH and glucose levels in real-time, offering multiparameter detection, integration of transmission and detection, and good biocompatibility. However, research on CQDs for imaging is in the very early stages. Further research is needed to extend the lifetime of the QDs. CQDs also use harmful UV light to excite the fluorescence, which needs further research to be used as a safe wearable glucose monitoring device [17,78,80].

#### 4.1.15. Raman Spectroscopy

**Figure 16:**
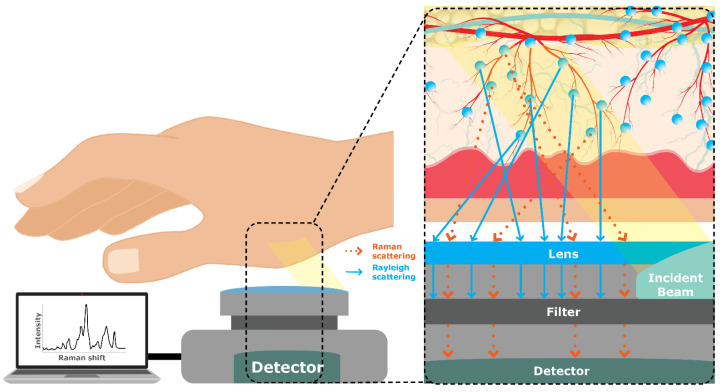
Raman Spectroscopy: illustration of intermittent device sensor.

Considered one of the most promising techniques, Raman Spectroscopy is an optical vibrational mode technique that can measure glucose through the skin and tissue using an NIR or visible laser beam (monochromatic light) range that excites the glucose molecules, scattering the light [25]. The light scattered is then collected and analyzed (Figure 16). The two types of scattered light are Rayleigh scattering and Raman scattering. Rayleigh scatters the light at the same frequency, whereas Raman scatters the light at different frequencies [14]. The difference between the scatterings of light from the excited molecules is known as Raman shift, and is the technique of choice by numerous devices, such as KnowU & Uband, Optical Glucose Monitoring System (OGMS), OptiScanner 5000, GlucoBeam, and Gluco Sense Diagnostics (see Section 4.6 on product classification).

The scattered light contains information about the molecular vibration/rotation in the sample, sending a signal to the receptors. The intensity of the glucose-specific Raman peaks is proportional to the concentration of glucose in the sample. Kang, Park [87] showed promising results in a novel non-contact fingerprint glucose detection device. While shining the light on an off-axis system and using a custom-made optical fiber bundle, the researchers were able to minimize probe instability, filter out specular Rayleigh reflection, and enable stable long-term measurements without tissue distortion.

#### 4.1.16. Surface-Enhanced Raman Scattering (SERS)

SERS is a variation of Raman Spectroscopy classified under nanoplasmonics that enhances the Raman light signal by several orders of magnitude [25,88], measuring the interaction of light scattered and absorbed by plasmons and molecules on the nanostructure [74]. There are two methods to enhance the signal in SERS: Chemical Enhancement (CE) and Electromagnetic Enhancement (EM) [73]. The CE mechanism is based on the chemical interaction between the analyte molecules and the surface of the metallic nanostructures used in SERS, while the EM mechanism is achieved through the interaction of light with plasmonic nanostructures, such as gold or silver nanoparticles, leading to a charge transfer providing a significant boost to the Raman signal intensity. Combining the two together, SERS provides exceptional sensitivity and selectivity for molecular detection. 

Pham, Seong [89] developed a SERS nanoprobe (SiO_2_@Au@Ag@4-MPBA) that increases the sensitivity of the detection of glucose. The researchers demonstrated that the 4-MPBA fraction of the nanoprobe reacts with the H_2_O_2_ generated in the presence of glucose by the GOx enzyme creating 4-MPheOH on the surface of probe, resulting in a variation of the SERS signal, which allows extrapolation to the concentration of glucose in solution with high accuracy, even at low concentrations. However, it is quite limited and in the early stages of research. Further research is needed on the nanostructure surface, optics, and advance machine learning processing to optimize SERS for a non-invasive glucose monitoring device [88]. 

Corcione, Pfezer [90] reported the application of an early-stage Surface-Enhanced Infrared Absorption (SEIRA) spectroscopy sensor combining the sensitivity of plasmonic systems and the specificity of standard infrared spectroscopy to glucose sensing analyzed with a Gaussian process regression machine learning model. The study suggested that this approach has the potential for glucose sensing and sensor calibration and warrants further research on Bayesian neural network analysis. 

#### 4.1.17. Surface Plasmon Resonance (SPR)

**Figure 17:**
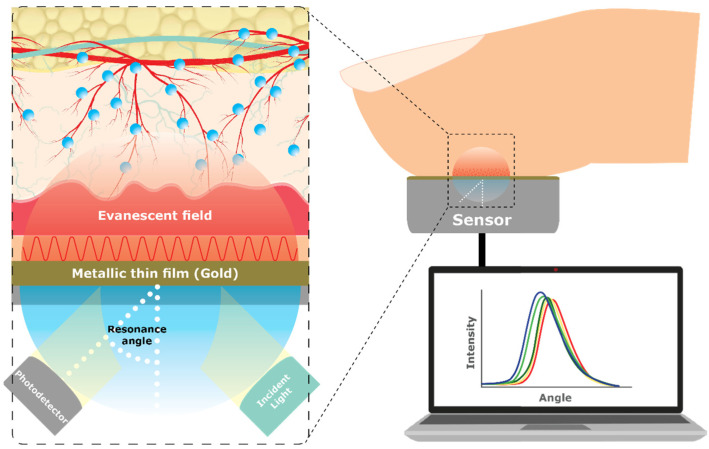
Surface Plasmon Resonance (SPR) technique illustrated in an intermittent finger sensor. The glucose affects the surface plasmon resonance causing shifts in the resonance angle and intensity of reflected light where the light wavelength is represented through color.

Similar to SERS, Surface Plasmon Resonance (SPR) is a plasmonic phenomenon. Yet, SPR occurs when an electromagnetic field interacts with a thin layer of metallic nanoparticles (Figure 17). The technique is based on the oscillation of electrons and involves shining light in the visible spectrum through a prism onto the metal layer, such as gold, and observing the reflected light to determine the resonance angle, which provides information on the glucose concentration [17]. The interaction with light creates a sensitive electric field that can detect changes in the surrounding material’s properties, such as its refractive index. By measuring the changes in the refractive index, including those caused by variations in glucose levels, researchers can track and analyze the shifts in location of resonance peaks. Plasmon resonance typically occurs at specific (visible or infrared) electromagnetic wavelengths of light and depends on the properties, size, shape, and composition of the metal nanostructures and surrounding medium [91]. Although research on SPR for glucose sensing has been focused on minimally invasive techniques that require external bodily fluids, such as urine [92], it can also be classified as a non-invasive alternative [17].

Kandwal, Nie [93] developed highly efficient and compact slow-wave Spoof Surface Plasmon Polariton (SSPP) end-fire antenna. The engineered phenomena, with high field confinement frequencies between 8 and 12 GHz and a sensitivity of 150 MHz resonance frequency shifts, were tested in vivo on five volunteers, demonstrating suitability for glucose sensing. Daher, Jaroszewicz [91] developed a novel Binary Photonic Crystal (BPhC) sensor with Infrared (IR) light, utilizing the refractive index changes in a defect cavity to shift the resonant peak, offering high sensitivity and a low detection limit for diagnosing diabetes. However, SPR’s limitations is that it is a very complicated technique; it is bulky, costly, has a long calibration time, and is highly sensitivity to motion, temperature, and sweat [25,69]. 

### 4.2. Electromagnetic and Electric Techniques

The substances within a tissue have different electrical properties and can act as either dielectrics, insulators, or conductors [94]. Glucose, when dispersed in water, acts as an insulator, and its levels can be indirectly detected by measuring the changes in the tissue’s electrical properties. The electromagnetic NIGST discussed are: Dielectric/Microwave Spectroscopy, Millimeter-Wave/Microwave (mmW/MW), Radio Frequency (RF) Spectroscopy, Bio-Impedance Spectroscopy, and Ultrasound Waves.

At low frequencies, such as below 100 MHz (radio frequency), electromagnetic waves can penetrate deeper into the layers of skin, fat, and muscle because they have longer wavelengths. However, as the frequency increases beyond 10 GHz (microwaves), the ability of the waves to penetrate muscle decreases significantly, while the penetration depths for skin and fat become shallower, which is an important limitation to consider for NIGST [95]. 

#### 4.2.1. Dielectric Spectroscopy or Impedance Spectroscopy

Often interchangeable with Impedance or Microwave (MW) Spectroscopy, Dielectric Spectroscopy is used for analyzing the complex magnetic permittivity of the skin or other relevant tissues by subjecting them to an electric field across a range of frequencies to determine variations in glucose concentration and analyzing information about their composition, structure, and changes in properties [95,96]. Glucose, being a polar molecule, can affect the dielectric properties of the surrounding medium. By analyzing the dielectric properties of a sample at different frequencies within the microwave range, it becomes possible to correlate the observed changes with glucose levels.

Despite unsuccessful devices from the past (e.g., PENDRA by Company: Pendragon Medical AG), DeepGluco™ by Alertgy (Melbourne, FL, USA) is currently developing a continuous wearable wrist device that uses Dielectric Spectroscopy to penetrate deeper into the tissue (see Section 4.6 on product classification) [95]. 

Omer, Shaker [97] developed a low-cost microwave sensor with a frequency range of 2.4–2.5 GHz, demonstrating promising results with high sensitivity in detecting glucose-level variations and the potential for personalized and accurate continuous glucose monitoring. 

Dielectric Spectroscopy can be affected by sweat and movement, as well as ambient, humidity, skin temperatures, and conductance, so it is important to measure these environmental and physiological parameters to improve its accuracy [98]. The technique can also be affected by water content, electrolyte concentration, and other factors associated with glucose levels. Combining personalized multisensor quasi-antenna arrays and machine learning-based signal processing, Hanna, Tawk [98] developed a novel wearable electromagnetic embedded holistic sensor system within sock garments, enabling wireless and continuous monitoring of glucose variations in the bloodstream with high accuracy. This multisensor monitored motion, Skin Conductance Response (SCR), skin temperature, external temperature, and humidity.

#### 4.2.2. Millimeter Waves and Microwaves (mmW/MWs)

While using regions of the electromagnetic spectrum, Millimeter Wave (mmW) and Microwaves (MWs) use specific frequency ranges that can be used in Dielectric Spectroscopy to penetrate deep tissue levels and accurately detect glucose molecules without the obstacles that are encountered with optical methods. Glucose molecules have characteristics that reflect, transmit, vibrate (in resonant systems), and bounce back (radar) depending on the frequency waves used, and correspond to the energy levels of the glucose for accurate measurement. The four methods of interactions of reflection (wide frequency range), transmittance (an advanced version of reflection in the wide frequency range), resonant perturbation (very narrow frequency range), and radar (far field range) spectrum can be used to infer the concentration of glucose in the sample [14]. 

The advantages of using mmW or MW sensing techniques is that they are highly sensitive with a fast response in real-time, are flexible, consume low power, are easy to manufacture, are robust, small in size, portable, and cost-effective, and measurements can be taken without precise alignment [99,100,101]. Spectral mmW occur between 30 and 300 GHz (or 1 and 10 mm), where, in particular, a W-band spectrometer (75–110 GHz) in the mmW spectrum has been shown to be an effective technique for in vivo glucose monitoring through skin tissue [102,103,104]. However, it is important to note that mmW and MW are not safe for continuous glucose monitoring, as repeat exposure could cause damage to the tissue due to their penetration depth [25]. 

Research has shown promise for glucose monitoring handheld devices for in vivo mmW measurements through the earlobe or finger web between 3 and 5 mm thick using silicon-loaded probes [105], through a compact finger slot reader or fingertip [40,96], and through the forearm [using bioheat transfer models;] [106], [using a near-field probe;] [107]. The studies reported that skin, fat, and blood have the most significant impact on the reflected signal, while muscle and bone have a negligible effect [107].

Similarly, Yang, Xiao [108] applied a Diffusion Limited Aggregation (DLA) fractal method to simulate soft tissue blood vessels and capillaries in a 3D earlobe, which was tested to reveal the frequencies of electromagnetic waves required to detect blood glucose levels. An ultra-wideband frequency range of 8–10 GHz with a sensitivity of 0.0198 dB per mg/dL was found to detect glucose level concentrations.

To go further in-depth within the interactions, the reflection method has been observed using a near-field coaxial conic probe to investigate its potential use in near-field diagnostics of blood glucose in a wide frequency range [109]. This study demonstrated that the probe could effectively monitor glucose concentration in phantoms of biological tissues with a resolution of 1 mmol/L (18 mg/dL) in the frequency range of 1.4–1.7 GHz with a signal-to-noise ratio of approximately 30 dB, confirming the feasibility of using this design for non-invasive glucose monitoring. 

The vibration method uses resonant frequencies to exhibit a peak response when interacting with glucose molecules and is influenced by changes in the tissues’ dielectric properties. Resonant frequency refers to the specific frequency at which a resonator is more stimulated, and changes in the material placed inside the resonator can alter this frequency and its characteristics, where minimum frequencies transmit and maximum frequencies reflect a resonator response [95]. A Split-Ring Resonator (SRR) and an active feedback loop has demonstrated high resolution and reliability in studies conducted by [110,111]. An SRR is a ring-shaped metal structure that exhibits resonant behavior when exposed to electromagnetic waves, causing it to absorb and re-radiate energy at certain frequencies, where the shift in resonant frequencies indirectly determines changes in glucose concentrations. However, various physiological and environmental factors can impact accurate and reliable measurements; one of which is temperature and movement. Jang, Park [101] used a temperature correction function (fluidic system) placed on the collarbone along with a complimentary SRR and found an increase in stability. By employing a single asymmetric SRR resonating at 7 GHz to create stability with the glucose measurement, Saleh, Ateeq [112] found high sensitivity. High sensitivity was also found with an advanced micro-fabrication technology and a mediator-free resonator [99]. More importantly, Yu, Rhee [113] displayed stability, consistency, and reliability with five microstrip antennas and a high resonant frequency band. The accuracy of resonator measurements can also be obtained with a single-step machine learning algorithm [114]. 

In a simulation study, Shaker, Smith [115] tested a retrofitted 60 GHz mm-W Soli alpha kit radar system (Google and Infineon based in Neubiberg, Germany) that detected changes in dielectric properties with promising results. They found that placing the transmit and receive antennas on opposite sides of the sample and adjusting the dielectric slab properties allowed for the detection and identification of glucose level changes, which has the potential of being implemented in a finger pulse oximeter wearable device. However, physiological variations in blood impacted the radar results and further research is needed to establish individual variation scaling methods and improve the mapping between the radar data and the actual glucose levels.

#### 4.2.3. Radio Frequency (RF) Spectroscopy

Similar to Dielectric Spectroscopy, mmW, and MW, Radio Frequency (RF) Spectroscopy involves analyzing the interaction between electromagnetic waves and tissue to extract glucose concentration [25]. However, RF Spectroscopy also uses MW in the regions of 0.1–20 GHz to measure the shifts in resonant frequencies using resonator sensors, such as Interdigital Transducers (IDT) or Stepped Impedance Resonators (SIR) [95,116]. Yunos, Manczak [116] used an RF sensor with IDT and SIR to remotely sense different glucose concentrations, showing a linear relationship between resonance frequency changes and glucose levels, indicating its potential for continuous non-invasive monitoring of diabetic and prediabetic patients. The concentration of glucose in the body affects the dielectric properties of the tissue, which in turn affects the RF signal, demonstrating a fast response time with a cost-effective technique [25]. 

Recently, GWave by HAGAR Tech (Hagar, Israel), a non-invasive smartwatch-based sensor, was presented as part of clinical trials with promising preliminary accuracy results (Zone A of the Clarke Error Grid (70–140 mg/dL range) with MARD of 7.1 (see Section 4.5 on developmental process). The GWave smartwatch uses RF waves and machine learning algorithms to analyze glucose levels, and sends readings via Bluetooth to a smartphone app, allowing for non-invasive continuous glucose monitoring (see Section 4.6 on product classification). 

Multiwavelength sensing using two modalities such as RF mmW with NIR transmission has also been found to significantly increase accuracy and sensitivity, while also supporting the capacity to be a wearable continuous monitoring device [68,117,118]. However, when combining sensors, this can increase the circuit space and comes with a risk of either error or interference. Nevertheless, the introduction of metamaterial-based sensors has shown promise for small-scale cell structures, demonstrating higher sensitivity to changes in glucose concentration compared with standard microstrip transmission line-based sensors that can be used for diagnosing hyperglycemia [112,119,120]. To overcome sensitivity limitations, Omer, Shaker [97] developed a portable fingertip prototype in a cost effective 2.45 GHz radar band, using an enhanced microstrip with an improved Complimentary Split-Ring Resonator (CSRR) configuration in a honey-cell structure.

#### 4.2.4. Bio-Impedance Spectroscopy (BIS)

Typically used for measuring body composition, Bio-Impedance Spectroscopy (BIS) involves applying an alternating electrical current to the skin and measuring the resulting voltage or conductivity. Where the current encounters resistance as it passes through different tissues and fluids, specifically at low frequencies of <5 kHz, the impedance (combination of resistance and reactance) can be measured [121]. 

**Figure 18:**
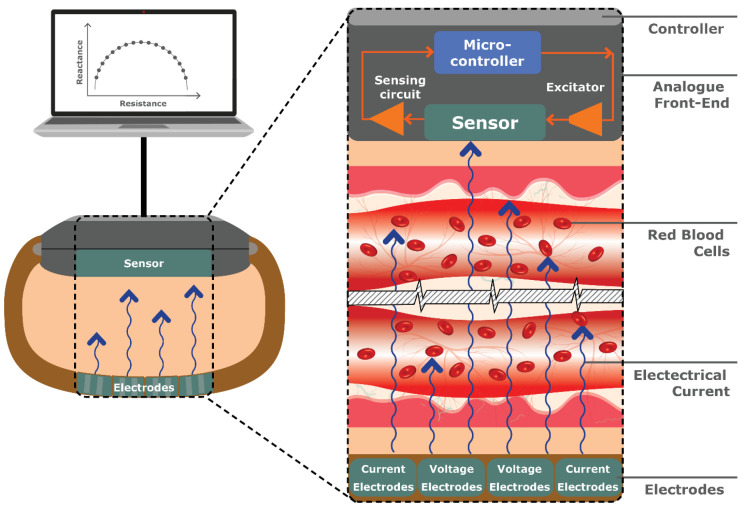
Bio-Impedance Spectroscopy technique illustrated in a continuous watch sensor.

BIS does not accurately predict the electrical impedance in the cells due to the cell-membrane capacitance [122]. Instead, BIS detects changes in the electrical properties of blood volume or Red Blood Cells (RBCs) in response to glucose variations, depending on the frequency setting (see Figure 18). 

Using Bio-Electromagnetic Resonance (BEMR), GlucoBand by Calisto Medical (Plano, TX, USA) has previously undergone trials for a continuous wristwatch connected to an armband; however, it has disappeared from the market [12]. Pedro, Marcôndes [122] used frequency ranges between 50 and 70 kHz alongside Effective Medium Theory (EMT) machine learning and found the estimated changes in glucose levels. However, the researchers stated that further research is needed to understand the ideal frequency range for measuring glucose levels. Similarly, Yen, Chen [41] demonstrated improved accuracy with the proposed combination of dual-wavelength PPG and BIS in the frequency range of 50 and 100 kHz placed between the index and middle fingers as well as under the wrists while using a Back Propagation Neural Network algorithm. However, it is important to consider the wrist circumference and wrist temperature, as well as body weight, as physiological parameters that can impact the accuracy of non-invasive glucose measurements [50]. 

BIS has been proven as a favorable technique for non-invasive glucose monitoring, being described as inexpensive, safe, small in size, reliable, and fast acting [121]. The main disadvantages of this technique are the sensor’s complexity and its instability with either sweat or movement [122]. A recent study developed a custom-fit biocompatible wearable ring and wrist device that collects bio-impedance data, while also monitoring skin temperature and movement to counteract the instabilities [121]. The novel prototype shows promise for an accurate, real-time, non-invasive, and continuous glucose monitoring device. 

#### 4.2.5. Ultrasound

This technology evaluates the propagation time (acoustic impedance) of ultrasound waves through a medium correlated with the glucose concentration in the body [14]. The speed of propagation is influenced by the glucose concentration, with higher concentrations resulting in faster propagation times.

Despite its limitations of high cost and sensitivity to temperature and pressure, the ultrasound’s ability to penetrate long distances into the tissue, its high sensitivity, and its indifference to skin color variations makes it ideal for combining with other techniques, in particular NIR Spectroscopy [25]. Kitazaki, Kawashima [123] proposed an ultrasonic-assisted MIR spectroscopic imaging method that creates an ultrasonic standing wave to generate a reflection plane at a depth of 100 μm from the sample surface, enabling non-invasive monitoring of glucose while overcoming the limitations of water absorption and high ultrasonic wave attenuation. However, ambient temperature can impact accuracy and needs consideration [14].

GlucoTrack’s by Integrety Applications Ltd. (Ashdod, MA, USA) and egm1000™’s by Evia Medical Technologies Ltd. (Weybridge, UK) earlobe devices for the intermittent estimation of glucose levels in type 2 diabetics use a combination of three different techniques, including ultrasonic waves, electromagnetic RF waves, and thermal spectroscopy (see Section 4.6 on product classification). Specifically, egm1000™ has Conformité Européene-mark approval and is currently available for purchase [124]. Combining techniques can significantly improve accuracy by either decreasing chances of errors or identifying and implementing correction for environmental or physiological parameters that affect accuracy [25], both of which benefit greatly from the advancements in machine learning techniques to improve accuracy.

### 4.3. Physiological Techniques

Some physiological parameters are directly or indirectly correlated with glucose levels. In a recent study, Wang, Mu [125] reported a 5 s 12-lead Electrocardiogram (ECG) with an IGRNet deep learning model analysis as effective in detecting and diagnosing prediabetes non-invasively. The study was based on the findings of previous studies reporting an impaired parasympathetic activity of the cardiac autonomic nerve function, observable as higher Resting Heart Rate (RHR) and longer P waves. Compared with blood glucose, ECG offers the advantage of not being influenced by other blood components, although is affected by other factors (such as BMI and gender).

Other techniques, based on physiological measurements such as blood pressure, humidity, skin temperature, ambient temperature, and galvanic skin response (not being a strong independent indicator of glycemic levels), are mostly described as trialed in combination with other techniques.

In a study by Bogue-Jimenez, Huang [126], a glucose-based smartwatch for continuous non-invasive glucose monitoring examined the use of multiple sensors, including optical, electromagnetic, and thermal techniques to measure up to 14 features, all measurable in a smartwatch-like wearable device and demonstrated to be related to blood glucose levels, including Heart Rate (HR), Skin Temperature (sTEM), Heat Flux (HF), Electrodermal Activity (EDA, also known as Galvanic Skin Response (GSR), Pulse Oximetry (SpO_2_), Systolic (SYS) and Diastolic (DIAS) blood pressures, Ambient Temperature (aTEM), and Ambient Humidity (aHUM). In a clinical trial in 2022, Hanna, Tawk [98] reported a continuous multimodality sensing system comprising humidity, skin temp, ambient temp, GSR, and EM hand and leg sensors, with a resulting 99.01% prediction rate of blood glucose levels.

In 2010, Australia-based company AiMedics Pty. Ltd. (Sydney, Australia), released HypoMon^®^ [127] for overnight monitoring of hypoglycemia in type 1 diabetics between the ages of 10 and 25 years. The system, made of a chest belt that non-invasively monitors electrocardiogram and skin impedance features, allowed to identify specific patterns of physiological responses to hypoglycemia, with a receiver containing interpretation algorithms. The system was then recalled in 2013 due to post-market rates of detection of hypoglycemic episodes lower than expected. Currently, the products NYSE:HIT by Hitachi Ltd. (Tokyo, Japan), GlucoGenius by ESERdigital (Hong Kong, China), and Gluco Quantum by Genki Vantage Limited (Hong Kong, China) are under development (see Section 4.6 on product classification).

A schematic output of all the articles presenting in vitro/in vivo data (*n* = 48) can be found in Supplementary Materials. The articles are divided by technique, highlighting advantages/disadvantages, machine learning application, sensor type, study phase (in vitro vs. in vivo), results, and future perspectives (A = in vitro, B = in vivo, C = larger cohort, D = machine learning application/expansion). 

Among the 48 articles included in the Supplementary Materials, the NIGST techniques explored the most were Millimeter Wave (mmw) and Microwaves (MW) (8 studies), followed by PPG (5 studies). A total of 21 studies were performed in vivo, with cohorts from 1 to 100, prospectively, [52] and 2914 cases, retrospectively [125]. The remaining studies were still in the in vitro phase. Thirteen articles have completed the in vitro/in vivo phase and warrant larger cohorts for the next phase of research: three for PPG, two for NIR, two for Dielectric Microwave, and one each for Millimeter/Microwave, MIR, BIS, SPR, and OCT.

### 4.4. Machine Learning

Machine Learning (ML), the core domain of Artificial Intelligence, is based on the use of software algorithms to identify patterns in large datasets. ML algorithms can be divided based on the level of human supervision into supervised, unsupervised, semi-supervised, and with reinforcement learning. Supervised ML algorithms can identify discrete output variables as categories or labels (classification analysis) or predict continuous values based on the input variables (regression analysis), although the two analyses can have some overlap. The several different techniques of machine learning are not the object of this paper and have been thoroughly discussed by Zhang and Zhang [128], Shokrekhodaei, Cistola [29], and Masson, Biggins [129]. 

To test the performance of machine learning models, internal and external validation can be used, respectively, validating the models with part of the original study or a new population [130]. The performance of each classification model can be expressed with the Jaccard Index, defined as the number of labels predicted correctly among the total predictions or AUC (Area Under the Curve), ROC curve (Receiver Operator Characteristic curve) generating probability values, where 0.8 identifies a strong classifier and 1 is a perfect classifier [29].

In the continuously evolving scenario of glucose sensing, machine learning represents a promising avenue for the improvement of detection and accuracy. The extensive and impenetrable load of data generated by glucose sensing techniques represents an optimal ground for machine learning application, with the potential of improving detection sensitivity [65]. 

Supplementary Materials Table S1 highlights the combination of machine learning techniques with glucose sensors encountered in this review. ML was applied to 25/48 studies, with a range of one (Chen, Lo [27] and Wang, Mu [125]) to eight techniques (Bogue-Jimenez, Huang [126]) trialed. PPG was the NIGST to which ML was most applied. All five articles on PPG had ML applied, compared with RF, CQD, and DRS, for which no ML was described by any article. The most used ML techniques were Partial Least Squares (PLS) regression (adopted in four articles), followed by Montecarlo simulation and Back Propagation Neural Network, adopted in three articles, and Support Vector Machine and XG boosts described in two articles.

To briefly explain these ML techniques, PLS is a regression method that creates new predictor variables (components) as linear combinations of the original variables, while considering the observed response values. MCS is based on random sampling of an actual amount to generate different scenarios of considered factors. BPNN is a multilayer feedforward network trained on an error back propagation algorithm [131]. XG boost is a supervised ML regression model based on a subsequential tree algorithm working in the presence of both categorical and numerical features with increased load capacity. Finally, SVM is an algorithm that learns by example to classify assigning labels to the study object [132].

The overall most promising results above the articles included in Supplementary Materials Table S1 were for studies investigating the use of PPG with the aid of ML: 100% of the measured values fell within CEG A–B, the lowest Standard Error of Prediction (SEP) was at 17.02 mg/dL, the MARD was between 4.4321 and 12/.19%, and the Pearson’s r was A 0.91 (see Supplementary Materials Table S1).

### 4.5. Developmental Process

**Figure 19:**
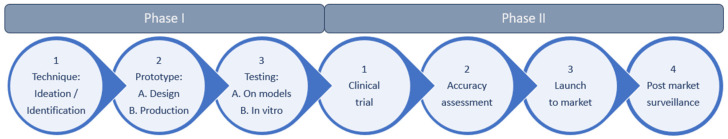
Glucose sensing production.

As with most medical equipment, the development of a glucose sensor encompasses two phases (Figure 19). 

Phase one concerns production and early testing. Once the glucose sensing technique to use is identified/ideated, a prototype of the device is designed and produced before testing it on models and/or in vitro. The device needs to be demonstrated as safe, suitable, accurate, and feasible for human use to meet the standards of the International Organization for Standardization (ISO)—namely, ISO 15197/2013 [133] for in vitro glucose monitoring instruments and self-monitoring glucometers—and be approved by the local authority (TGA (Therapeutic Good Administration in Australia), FDA (Food and Drug Administration in U.S.A.), and/or EMA (European Medicine Agency in EU)) before proceeding to phase two of validation.

These standards provide guidance on minimum accuracy requirements on the device, as well as on labeling, user manuals, and training materials. In terms of accuracy, at least 95% of the results must fall within ±15 mg/dL of the reference method for blood glucose concentrations below 100 mg/dL and within ±15% for concentrations greater than or equal to 100 mg/dL. 

During the clinical trial, the system’s accuracy in measuring blood glucose levels is confirmed in vivo with either MARD, CEG (Clarke Error Grid), or PEG (Parkes Error Grid) [46]. MARD is a percentage of errors from the measured value compared to a reference value. The error grids, instead, measure the accuracy of non-invasive glucose monitors, qualitatively comparing the result of the sensor versus the reference method. The main difference between CEG and PEG is that the latter differentiates between type 1 diabetes and type 2 diabetes (Table 3). 

Once the sensor is released to the market, its performance continues to be surveyed in the post-market analysis.

### 4.6. Product Classification

**Figure 20:**
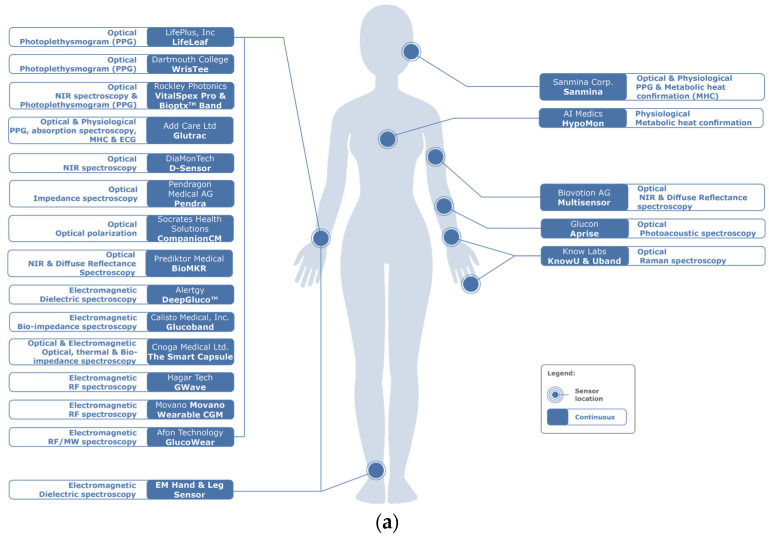

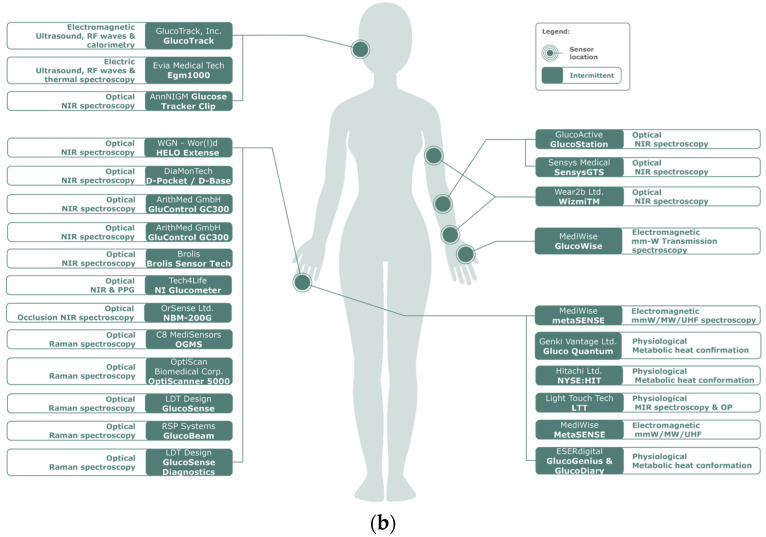
(**a**) Continuous glucose sensing product site. (**b**) Intermittent glucose sensing product site.

Based on the information provided in the literature and Google searches, the glucose sensing products reviewed in this paper were classified as either continuous (Figure 20a) or intermittent (Figure 20b) based on the sensor site (for more information, please review [12,14,94]). A more schematic representation of all the products can be found in Supplementary Materials Table S2. 

Among the CGM devices, PPG was the technique most used (3); however, among the intermittent glucose monitoring techniques, the one most frequently adopted was NIR spectroscopy (10), followed by Raman spectroscopy (5). Despite various attempts and developments in non-invasive glucose monitoring devices, challenges with accuracy, sample collection, and environmental and physiological variability have led to the withdrawal or ruling out of certain technologies, such as GlucoWatch and Pendra [14]. Specifically, Pendra was found in a post-market analysis to not accurately estimate blood glucose levels for all users and required a complex calibration procedure [126].

Of the 47 products found for non-invasive glucose monitoring, there are currently no FDA-approved products on the market. Four products have received the Conformité Européene (CE) mark of approval that meets European safety, health, and environmental standard requirements and are currently being sold in Europe, including GlucoTrack (Company: Integrity Applications, Ltd., Rutherford, NJ, USA), GWave by HAGAR Tech (Hagar, Israel), Glutrac by Add Care, Ltd. (Edmonton, AB, Canada), and Egm1000 by Evia Medical Technologies, Ltd. (Weybridge, UK). Glutrac also meets the National Medical Products Administration (NMPA) standards, now known as China Food and Drug Administration, and is simultaneously sold in China. 

Consistent with other products in development, Glutrac and GWave are classified as continuous wearable devices monitoring glucose levels on the wrist. 

The wrist, arm, ankle, and chest are optimal body locations for continuous wearable devices, whereas the fingertip, palm, and earlobe can provide intermittent point-of-care glucose sensing. The search for a truly non-invasive and reliable glucose monitoring solution continues.

## 5. Discussion

To the best of our knowledge, this is the most recent comprehensive review on non-invasive glucose sensing techniques written from healthcare and industrial design perspectives. The additional novelty of this study includes the exploration of a machine learning application for each technique, as well as of products in the development/validation phase.

The classification of glucose sensing techniques is not uniform in the literature. Within different reviews, some techniques that use saliva, sweat, and tears are reported as either non-invasive or minimally invasive. Our classification is based on non-invasiveness in optical, electromagnetic, and physiological techniques.

Of the techniques included in this review, PPG was the most investigated in vitro within the past five years (from 2018 to 2023) and resulted in having the most extensive application of machine learning. Of the developed products, none have been approved in all markets (EMA, ETG, etc).

To identify the most promising techniques, we considered the WHO REASSURED criteria, which consider real-time connectivity and ease of specimen collection, but also if the test is affordable, sensitive, specific, user-friendly, rapid and robust, equipment free or simple, environmentally friendly, and deliverable to end-users [134]. The REASSURED criteria represent an optimal tool to identify NIGST worthy of further development in the context of the diabetes epidemic [135]. As the diabetes trend is currently rising, particularly in Low- and Middle-Income Countries (LMIC) due to a sudden increase in availability of food after years of malnutrition and starvation, the need of a cost-effective and user-friendly glucose sensing technique is becoming critical [136]. 

Based on the REASSURED criteria, the studies and products that resulted warranting further research and testing were based on PPG and NIR. Both techniques are described as low cost, offering high accuracy with minimal occupied space and potential for continuous NIGST. While PPG is sensitive to motion and movement [38], NIR is sensitive to tissue thickness, temperature, skin tone/melanin, tissue substances, and ambient light intensity [14]. Further research is needed on a multisensory approach to counteract these limitations, Zhang, Liu [26].

NIGST development can be considered expensive, and this may seem to be in contrast with the need to prioritize the diagnostic coverage in LMIC. If considering the additional cost of each DM diagnosis, which could be avoided with refined diagnosis and management of the different types of diabetes, the investment in NIGST may be appropriate and shows promise in reducing the burden of DM in the healthcare system [137]. A cost analysis of all the techniques and devices is not included in this review due to the paucity of information in the literature, and is warranted for the most promising techniques identified within it.

Our study identifies novel promising techniques and underlines how machine learning can boost accuracy and reliability. Machine learning is reported to be a precious tool in augmenting the sensitivity and accuracy of PPG and NIR techniques. Furthermore, ML could be considered to stratify personal risk for the disease and develop a tailored management plan. This is particularly important for diabetes, as it is considered more than ever a dynamic condition based on personal predisposition, lifestyle, and daily environment [138]. Given the long training time for complex validation, further research is needed to verify the role of machine learning in optimizing NIGST [37,139]. 

## 6. Conclusions

NIGST could represent the key to contain the diabetes epidemic by addressing the low acceptability and sensitivity of the current DM diagnostic and management methods. Applying machine learning to NIGST could be the key to optimizing glucose sensing for Diabetes Mellitus diagnosis and management.

Considering the WHO REASSURED criteria, the NIGST was the most explored, with a higher application of ML techniques, product development, and optimal accuracy, where PPG was among the continuous and NIR/Raman Spectroscopy was among the intermittent techniques. Further research is warranted to validate their use in the different subgroups of diabetic patients with larger in vivo research, machine learning application, costs analysis, and acceptability surveys.

## Figures and Tables

**Table 1:** Techniques and corresponding band, frequency, and wavelength.

Technique	Band	Frequency	Wavelength
Radio Frequency (RF)	Low Frequency (LF)	30–300 kHz	10–1 km
	Medium Frequency (MF)	300–3000 kHz	1 km–100 m
	High Frequency (HF)	3–30 MHz	100–10 m
	Very High Frequency (VHF)	30–300 MHz	10–1 m
	Ultra-High Frequency (UHF)	300–3000 MHz	1 m–1 cm
Microwave (MW)	Super-High Frequency (SHF)	3–30 GHz	10–1 cm
Millimeter Wave (mmW)	Extremely High Frequency (EHF)	30–300 GHz	1 cm–1 mm
Terahertz (THz)	Terahertz Frequency (THF)	300 GHz–10 THz	3 mm–30 µm
Optical	Infrared (IR)	3–500 THz	100 µm–600 nm
	Visible	500–1000 THz	600–300 nm

**Table 2:** Different interactions of light and electromagnetic waves with tissue and glucose.

Aspect	Reflection	Scattering	Absorption	Transmission
Interaction Mechanism	Directed onto the tissue surface and reflects off it.	Directed into the tissue, emerges at different angles.	Components absorb specific wavelengths.	Passage of light or waves through tissue.
Interaction with Glucose	Glucose affects the reflected light or radio waves.	Glucose cause changes in tissue refractive index and scattering properties.	Glucose molecules absorb waves or light at certain wavelengths, affecting the tissue light absorption.	Glucose molecules absorbing light change the vibrational mode, decreasing the intensity of transmitted light.
Obtained Information	Angle of reflection.	Angle of scattering.	Concentration of glucose in sample.	Absorption patterns, tissue transparency, and decrease in transmitted light/wave intensity.
Techniques	Optical Polarimetry (OP), Diffuse Reflectance Spectroscopy (DRS), and Ultrasound Waves.	Optical Coherence Tomography (OCT), Scattering/Occlusion Spectroscopy, Raman Spectroscopy, and Ultrasound Waves.	Photoplethysmography (PPG), Near Infrared (NIR) Spectroscopy, Mid-Infrared (MID) Spectroscopy, Far-Infrared (FIR) Spectroscopy, Photoacoustic Spectroscopy, Fluorescence, Radio Frequency (RF) Spectroscopy, Millimeter Waves (mmW), Microwaves (MW), NIR/MID Absorption Spectroscopy, and Bio-Impedance Spectroscopy.	Photoplethysmography (PPG), Near Infrared (NIR) Spectroscopy, Mid-Infrared (MID) Spectroscopy, Far-Infrared (FIR) Spectroscopy, Radio Frequency (RF) Spectroscopy, Millimeter Waves (mmW), Microwaves (MW), and Bio-Impedance Spectroscopy.
Challenges	Variability in skin properties (e.g., skin color, texture) can affect measurements, ambient light interference can impact accuracy and signal interference.	Complex scattering patterns can be difficult to interpret, and the depth-dependent effects of scattering impact accuracy.	Overlapping absorption by various tissue components, variations in tissue composition, and calibration challenges can affect accuracy.	Scattering effects can alter the light paths/radio waves and proper sample handling. Interference can impact results, rendering it a complex interaction.

**Table 3:** Accuracy of glucose sensing techniques. Adapted from Alsunaidi, Althobaiti [46].

Risk Zones	CEG Analysis	PEG Analysis
Zone A	Clinically valid treatment	No effect on clinical treatment
Zone B	Clinically uncritical treatment	Mild effect on clinical treatment
Zone C	Unnecessary treatment	Possible effect on clinical treatment
Zone D	Dangerous: fails to diagnose and treat	Serious medical risks
Zone E	Extremely dangerous: leads to wrong treatment	Dangerous consequences

## Data Availability

The data presented in this study are available in Supplementary Materials.

## Supplementary Materials

The following supporting information can be downloaded at: https://www.mdpi.com/article/10.3390/s23229130/s1, Table S1: studies in vitro/in vivo; Table S2: Continuous and Intermittent non invasive glucose sensing products. Reference [139] is cited in the supplementary materials.
