# Supplementary material for: Non-Invasive Glucose Sensing Technologies and Products: A Comprehensive Review for Researchers and Clinicians
Source: Sensors (Basel). 2023 Nov 12;23(22):9130. doi: 10.3390/s23229130 (PMC10674292; doi:10.3390/s23229130)
Supplement: Supplementary file 1 [file sensors-23-09130-s001.zip › sensors-2675903-supplementary.pdf]

Table S1

TECHNIQUE/ CATEGORY	STUDY OBJECT	ADVANTAGES	DISADVANTAGES	STUDIES	MACHINE LEARNING	PRODUCTS WITH PHASE	SAMPLE	FINDINGS	FUTURE PERSPECTIVES
					**OPTICAL TECHNIQUES**				
**Optical Coherence Tomography (OCT)**	Reflection of light by tissue with interference from glucose	Unaffected by heartrate, blood pressure, osmolytes	High sensitivity to movement and skin temperature	Reference [27]	Mueller Model Algorithm	Fingertip device	In vitro + In vivo (4 + 3)	R2 ¼ 0.5993, Avg SD of D among 5 sets of measurements 0.027, SD of the γ and Δ at five times: 0.302 deg and 0.045	C
				Reference [28]	N/A	Low magnification objective lens	In vitro	Improved accuracy	A
**Optical Polarimetry (OP)**	Rotation/absorption energy of polarized light plane reflected from glucose	Accuracy, high resolution, can be small in size	Low sensitivity with low glucose levels, sensible to albumin levels and lower sensitivity with dark skin	Reference [13]	Partial Least Squares (PLS) regression	Noncontact glucometer device in vitro + in vivo (animals)	In vitro+ in vivo (4 rabbits)	Mean differences of 8 mg/dl and 29.2 mg/dl for the in vitro and in vivo measurements between aqueous and blood glucose	B
				Reference [31]	Boosted trees regression learning model	Palm and finger sensor compact and low cost (i.e., USD <250) with off-the-shelf hardware	In vivo (41 T2DM + 9 C)	89% in CEG A, ARD 10% with CGM – no training or calibration needed	A+C (in vitro: smaller size and decreased costs)
				Reference [32]	N/A	Spatial Polarization Modulation System (SPMS)	In vitro	Resolution of 100 mg/dl	B
				Reference [33]	Partial Least Squares (PLS) regression algorithm	Broadband polarimeter	In vitro	More precise prediction of glucose concentration in the presence of albumin	A
**Photoplethysmography (PPG)**	Amount of LED light transmitted/reflected measured with photodetector	Low cost ear canal sensing has stable temperature, constant pressure, consistent positioning,	Sensible to blood circulation and movement	Reference [39]	Support Vector Machines (SVM), Gaussian process regression, kernel approximation	in-ear device equipped with a low-cost pulse oximeter	In vivo (1 preDM, 1 T1DM, 1T2DM, 1 C)	82% of the BGLs in CEG A, 100% CEG A -B	C

		universal fit, and cost-effectiveness			models, ensemble models, Neural Networks (NNs)				
				Reference [36]	Partial Least Squares (PLS) regression	Smartphone video-based technique	In vivo: 52 subjects	lowest Standard Error of Prediction (SEP) at 17.02 mg/dL	C
				Reference [41]	Back-Propagation Neural Network (BPNN)	dual-wavelength PPG combined with bioelectrical impedance	In vivo: 40 subjects	mean squared error of 40.736, a root mean squared error of 6.3824, a mean absolute error of 5.0896, a mean absolute relative difference of 4.4321%, and a coefficient of determination (R Squared, R2) of 0.997,	C + D
				Reference [35]	Monte Carlo S and XGBoost	Fingertip model	A. In vitrofinger mode—1581 samples<br>A. In vivo—35 participants	Pearson's r = A 0.91 model/B 0.85, coefficient of determination (R^2) = A 0.83/B 0.68	A +C<br>(in vitro: variation in layer thickness)
				Reference [42]	Singular Spectrum Analysis (SSA)	Wearable device	In vivo: 8 volunteers	87.06% of data on zone A of CEG, and MARD of 12.19%	C
**Near-Infrared (NIR) spectroscopy**	Reflection of light	Considered one of the most effective techniques,<br>low cost, compact, and high penetration level,	Low accuracy and absorption pattern, sensitive to tissue thickness, temperature, skin tone and melanin, substances in the tissue (fat, protein,	Reference [47]	Linear Partial Least Square Regression (PLSR) with the nonlinear Stacked Auto-Encoder (SAE) deep neural network.	light source, optical switch, optical fiber bundle and optical fiber probe, detector, and thermostat	In vivo: 19	97.96% in CEG A	C

			and water), and ambient light intensity	Reference [43]	Monte Carlo simulations	Inner lip— calibration method	In vivo: 1	“Extremely positive results”	A+C (in vitro: delay between the glucose concentration in blood and skin tissue)
				Reference [49]	NIL	NIR spectroscopy alongside SpO2 and heartrate in a compact fingertip sensor	In vivo: 10 (2 DM)	USD 15.6 expense average deviation for glucose was found to be 2.54%	C
				Reference [19]	Monte Carlo simulation	Dual-channel measurement for glucose and noise signal for different skin colors	In vitro: finger models with different melanin concentration	Increased accuracy, better signal-to-noise ratios in darker skin	B
**Mid-Infrared (MIR) spectroscopy / Optical**	Narrow band light transmitted/reflected by vibration of glucose molecules	More distinct absorption, up to 100 μm penetration depth with no irritation/discomfort	High cost	Reference [52]	Xgboost algorithm	Developmental phase of handheld device, D-Base (Company: diomontech AG)—trial	100 human samples (41 diabetic and 59 healthy)	Less than 1% of the data outside of the highest accuracy zones	C
**NIR/MID absorption spectroscopy**	Combination of aspects from Near-Infrared (NIR) and Mid-Infrared (MID) light	More comprehensive spectrum of absorption by glucose molecules in body tissues	See NIR/MID	Reference [29]	K nearest neighbor, decision tree, SVM, multiple linear regression, artificial NN	Multiwavelength measurement in the Visible (VIS) and NIR range	IN VITRO distilled water solutions—4000 total test	99.8% in CEG A with SVM	B
**FAR-Infrared Spectroscopy (Terahertz Spectroscopy)**	Vibrational and rotational transitions of glucose	N/A	Susceptible to strong light absorbance by water	NIL STUDY ANALYZED					

**Terahertz-Time Domain Spectroscopy (Thz-TDS)**	Time delay and magnitude of the reflected or transmitted short pulses light as they interact with glucose	Safe<br>Offers labels free sensing	Significant obstacle in developing label-free Terahertz (thz) sensors that can detect analytes with high sensitivity	Reference [57]	Back Propagation Neural Network (BPNN)	Waveguide probe sensor unit	IN VITRO PHANTOM	Low-cost, accurate, and highly sensitive	A+B<br>(conceptual/in vitro earlobe device)
				Reference [58]	N/A	S-shaped complementary resonators	In vitro	Good sensitivity, compact	B
**Time of Flight (TOF)**	Additional capability from (Thz-TDS) to sweep through a wide range of frequencies	More detailed information about the material's properties	As above	NIL STUDY ANALYZED					
**Thermal Emission Spectroscopy (TES)**	Infrared radiation emitted by the body as influenced by glucose levels	No penetration of light/wavelengths or calibration needed require	Sensible to temperature, movement, tissue thickness. Limited real-time capability	NIL STUDY ANALYZED					
**Scattering/Occlusion Spectroscopy**	Interaction of light waves through matter	Highly sensitive, safe, minimal interference, convenient, allows for real-time monitoring	Sensible to variations in tissue protein, fat, red blood cells aggregation, blood osmolality, skin characteristics	NIL STUDY ANALYZED					
**Photoacoustic Spectroscopy (PAS)**	Heat energy creating a sound (acoustic) wave created with heat energy released by glucose on light absorption	Rapid response time, high sensitivity and selectivity, and is very precise	Water, fat, proteins, and other substances can increase light absorption, skewing results of glucose concentration	Reference [66]	Ensemble classification model	A single-wavelength MIR QCL light source with frequency modulation wavelengths of 10 to 30 khz	In vitro phantoms	Detection of glucose for the entire range of interest of healthy/diabetic pt	B
				Reference [64]	N/A	T-type opened resonator cells with frequency of 25 to 52 khz	Not trialed—developed only	The detection of Photoacoustic (PA) signals can be enhanced	A
**Diffuse reflectance**	Reflected light to analyze absorbance	Real-time monitoring capability and applicability to	Interference from other tissue components and temperature drifts	Reference [68]	N/A	NIR DRS prototype with a laser with two detector photodiodes	In vitro	Miniaturization not possible	A+B<br>(in vitro: calibration)

**spectroscopy DRS**	coefficient parameters	different tissue thicknesses	causing a delay and instability	Reference [67]	N/A	NIR wavelengths of 900 – 1300	In vitro: 26 phantom	Based on CEG measuring up to 18mM	A+B (in vitro: calibration)
**OPTICAL - NANOTECHNOLOGIES**									
**Plasmon-Enhanced (PEF) Fluorescence**	Fluorescence signal from probes/molecules h	potential for a cost-effective non-invasive	Photobleaching for fluorescence	NIL STUDY ANALYZED					
**Carbon Quantum Dot (CQD) Fluorescence**	Changes to fluorescence emission based	low toxicity/cost, high stability, eco-friendly, biocompatible, versatility in size, applicable to skin patches/temporary tattoos, multiparametric evaluation	CQDs use harmful UV light to excite the fluorescence Research at early stages	Reference [86]	N/A	Excitation wavelength of 360 nm	In vitro	Hydrogel film more stable than aqueous solution but as sensitive	A+B
				Reference [139]	N/A	Hydrogel optical fiber fluorescence sensor for Ph and glucose levels	In vitro	Simultaneous continuous monitoring of pH and glucose levels in real-time	B
**Raman Spectroscopy**	Difference between scattering lights from excited glucose		Probe instability Tissue distortion	Reference [88]	Multiple Linear Regression (MLR)	Non-contact fingerprint glucose detection device with optical fiber bundle	In vitro In vivo—pigs ears	Pearson correlation coefficient, R = 0.90 on average	B
**Surface-Enhanced Raman Scattering (SERS)**	Interaction of light scattered and absorbed by plasmons and molecules on the nanostructure	Exceptional sensitivity and selectivity for molecular detection	Early researching phase, costs	Reference [90]	N/A	SERS nanoprobe (SiO2@Au@Ag@4-MPBA)	In vitro	high accuracy, even at low concentrations	A+B
				Reference [91]	CascadeForward Neural Network	Surface-Enhanced Infrared Absorption (SEIRA) spectroscopy sensor	In vitro: distilled water solutions—4485 total test	Potential glucose sensing and sensor calibration	B + D
**Surface Plasmon Resonance (SPR)**	Resonance angle of light through a prism onto a metal layer, such as gold	Highly efficient and compact	Very complicated, bulky, costly, long calibration time, high sensitivity to motion/temperature/sweat	Reference [94]	N/A	Slow-wave Spoof Surface Plasmon Polariton (SSPP) end-fire antenna	In vivo: 5 subjects	Near-linear correlation between the glucose values and resonance shifts	C
				Reference [92]	N/A	Novel binary photonic crystal (bphc) sensor with infrared (IR) light	In vitro	High sensitivity, and low detection limit	B
**ELECTRIC/ELECTROMAGNETIC TECHNIQUES**									

**Dielectric/Microwave (mw) Spectroscopy**	Changes in magnetic permittivity (electric field)	Enables wireless and continuous monitoring of glucose variations in the bloodstream with high accuracy	Sensible to sweat/movement/ ambient humidity/ skin temperatures/ water content/ electrolyte concentration	Reference [98]	PCA	Low-cost microwave sensor with frequency range 2.4 – 2.5 ghz	In vitro<br>In vivo: 2	No delay compared with BGM, low cost, small size	C
				Reference [99]	Gaussian Process Regression (GPR)	Wearable multi-sensor system of blood glucose, motion, skin conductance response/temperature, external temperature, humidity	In vivo: rats, pigs, 28 humans (18 diabetic, 10 healthy)	Clinical accuracy of continuous glucose monitoring of 99.01%	C
**Millimeter Wave (mmw) and Microwaves (MW)**	Reflection, transmittance, vibration (resonant perturbation), and re-bounce (radar) of waves encountering glucose	Highly sensitive, real-time readings, flexible, consumes low power, easy to manufacture, robust, small in size, portable, cost-effective, nil precise alignment needed	Not safe for continuous glucose monitoring as repeat exposure could cause damage to the tissue. Sensible to physiological variations in blood	Reference [106]	Complex-Valued Neural Network (CVNN)	Measurements through the earlobe or finger web between 3 and 5 mm thick using silicon-loaded probes	In vitro<br>In vivo	Silicon loading at the probes enhances the CVNN ability	C
				Reference [40]	N/A	Compact finger slot reader or fingertip	In vitro	Cost-effective, compact, non-ionizing in nature, and convenient	B
				References [107, 108]	N/A	Forearm (near-field and bioheat transfer models)	In vitro	High accuracy	B
				Reference [109]	N/A	Earlobe probe with Diffusion Limited Aggregation (DLA) fractal method	In vivo	Feasibility	B
				Reference [110]	N/A	Near-field coaxial conic probe using reflection method in the frequency range of 1.4 – 1.7 ghz	In vitro: phantoms	Resolution of 1 mmol/L (18 mg/dl)	B
				Reference [116]	N/A	Retrofitted 60 ghz mm-W Soli alpha kit radar system	In vitro	Potential for high accuracy but in early phases	A+B
				Reference [113]	N/A	Single asymmetric SRR resonating at 7 GHz	In vitro	High sensitivity	B
				Reference [114]	N/A	Five microstrip antennas and high	In vitro	Stability, consistency, and reliability	B

						resonant frequency band			
**Radio Frequency (rf)**	Shifts in resonant frequencies	Cost efficient, compact, seamlessly connecting to Bluetooth/smartphones	Mathematical model complexity	Reference [117]	N/A	Interdigital Transducers (IDT) or Stepped Impedance Resonators (SIR)	In vitro	Linear relationship between resonance frequency changes and glucose levels	B
				Reference [98]	N/A	Portable fingertip prototype at 2.45 ghz radar band	In vitro	Cost effective	B
**Bio-Impedance Spectroscopy (BIS)**	Voltage changes to alternating electrical current to the skin	Inexpensive, safe, small in size, reliable, and fast acting.	Wrist circumference and wrist temperature, as well as body weight as physiological parameters which can impact the accuracy + sensor's complexity, and is unstable with either sweat or movement	Reference [123]	Effective Medium Theory (EMT)	Simplified physical model for blood electrical conductivity	In vitro	maximum error of 5.2%, early phase	A+B
				Reference [41]	Back-Propagation Neural Network	Combination of dual-wavelength PPG and BIS in the frequency range of 50 and 100 khz between the index and under the wrists	In vivo: 40 subjects	MARD of 4.4321% within the clinically accurate region A in the Clarke Error Grid analyses	C
				Reference [112]	N/A	Custom-fit, biocompatible, wearable ring and wrist device for bio-impedance, skin temperature, and movement	In vivo: 14 T2DM	Mean Absolute Relative Difference (MARD) of 17.9, 99% values CEG A-B	C+D
**Ultrasound (US)**	Propagation time of US waves through a medium (propagation time or acoustic impedance)	Ability to penetrate long distances into the tissue, high sensitivity. Not sensible to skin color variations	High cost, sensitivity to temperature and pressure	Reference [124]	N/A	Ultrasonic-assisted MIR spectroscopic /OCT imaging method	In vitro	Potential for non-invasive monitoring of glucose	C+D
**PHYSIOLOGICAL TECHNIQUES**									

**Cardiac features**	Higher Resting Heart Rate (RHR) and longer P waves when cardiac autonomic nerve function impaired by glucose	Not being influenced by other blood components	Affected by other factors (such as BMI and gender)	Reference [126]	Deep Neural Network	IGRNET = 5 seconds 12-lead Electrocardiogram (ECG)	In vivo (retrospective) : 2914 cases	Diagnostic accuracy 0.781, AUC is 0.777	C (prospective)
				Reference [127]	Linear Regression (LR), Support Vector Regression (SVR), K Nearest Neighbors Regression (KNN), Decision Trees Regression (DTR), Bagging Trees Regression (BTR), Random Forest Regression (RFR), Gaussian Process Regression (GPR), and Multi-layer Perceptron Regression (MLP or NNR)	Glucose-based smartwatch with optical, electromagnetic, and thermal sensors to measure up to 14 features among which HR, blood pressure	In vivo (retrospective+ prospective)	No accurate enough prediction	A

A = in vitro, B = in vivo, C = larger cohort, D = Machine Learning application/expansion

Table S2

Device	Company	Method	Technique	Site	Continuous / Intermittent
**Glucoband**	Calisto Medical	Electromagnetic	Bio-Impedance Spectroscopy (Bio-Electromagnetic Resonance (BEMR™)	Wrist	C
**GlucoTrack**	GlucoTrack, Inc.*	Electromagnetic	Bio-Impedance Spectroscopy	Wrist	I
**DeepGluco™**	Alertgy	Electromagnetic	Dielectric Spectroscopy	Wrist	C
**EM Hand and Leg Sensor**		Electromagnetic	Dielectric Spectroscopy	Wrist, hand, leg	C

**Multisensor**	Solianis Monitoring AG / Biovotion AG	Optical	Diffuse Reflectance Spectroscopy (DRS) and NIR Spectroscopy	Arm	C
**BioMKR**	Diktor Medical	Optical	Diffuse Reflectance Spectroscopy (DRS) and NIR Spectroscopy	Wrist	C
**Pendragon Pendra**	Pendragon Medical AG	Electromagnetic	Impedance Spectroscopy	Wrist	C
**HypoMon**	AI Medics	Physiological	Metabolic heat conformation	Chest	C
**GlucoGenius**	ESERdigital	Physiological	Metabolic heat conformation	Finger	I
**NYSE:HIT**	Hitachi Ltd.	Physiological	Metabolic heat conformation	Fingertip	I
**Gluco Quantum**	Genki Vantage Limited	Physiological	Metabolic heat conformation + **	Fingertip	I
**LTT**	light touch technology	Optical	MIR Spectroscopy/optical parametric oscillation	Finger	I
**metaSENSE**	MediWise	Electromagnetic	mmW/MW/UHF Spectroscopy	Finger	I
**GlucoWise**	MediWise	Electromagnetic	mm-Wave Transmission Spectroscopy	Hand	I
**WizmiTM**	Wear2b Ltd	Optical	NIR Spectroscopy	Arm, Wrist	I
**Glucose tracker clip**	AnnNIGM	Optical	NIR Spectroscopy	Earlobe	I
**Brolis Sensor Technology**	Brolis	Optical	NIR Spectroscopy	Finger	N/A
**GluControl GC300**	ArithMed GmbH and Samsung Fine Chemicals Co. Ltd	Optical	NIR Spectroscopy	Finger	I
**HELO Extense**	World Global Network (WGN) - Wor(l)d	Optical	NIR Spectroscopy	Finger	I
**GlucoStation**	GlucoActive	Optical	NIR Spectroscopy	Forearm	I
**Sensys GTS**	Sensys Medical Inc	Optical	NIR Spectroscopy	Forearm	I
**D-S/P*****	DiaMonTech	Optical	NIR Spectroscopy	Wrist / Finger	C / I
**TouchTrack Pro**		Optical	NIR Spectroscopy		
**VitalSpex Pro & Bioptx Band**	Rockley Photonics	Optical	NIR Spectroscopy and Photoplethysmography (PPG)	Wrist	C
**NBM-200G**	OrSense Ltd.	Optical	NIR Spectroscopy (Occlusion)	Finger	I
**No-Invasive Glucometer**	Tech4Life	Optical	NIR/PPG	Finger	I
**Companion CM/SR/LS *** **	Socrates Health Solutions	Optical	Optical polarization	Wrist/ Earlobe/ Finger	C / I / I
**The Smart Capsule™**	Cnoga Medical Ltd.	Optical & Electromagnetic	Optical, thermal, bioimpedance signals	Wrist	C
**Aprise**	Glucon	Optical	Photoacoustic Spectroscopy	Forearm	C
**WrisTee**	Dartmouth College	Optical	Photoplethysmography (PPG)	Wrist	C
**GlucoDiary**	ESERdigital	Optical and Physiological	photoplethysmography (PPG) and Metabolic heat conformation	Finger	I

**Sanmina**	Sanmina Corp.	Optical	Photoplethysmography (PPG) and Metabolic heat confirmation	Ear canal	C
**LIFELEAF**	LifePlus, Inc.	Optical	PPG	Wrist	C
**Glutrac**	Add Care Ltd	Optical	PPG, absorption spectroscopy, metabolic heat conformation, and ECG	Wrist	C
**KnowU & Uband**	Know Labs	Optical	Raman Scattering	Finger, wrist	C
**OGMS*** **	C8 MediSensors	Optical	Raman Spectroscopy	Finger	I
**OptiScanner 5000**	OptiScan Biomedical Corporation	Optical	Raman Spectroscopy	Finger	I
**GlucoBeam**	RSP Systems	Optical	Raman Spectroscopy	Finger / Palm	I
**GlucoSense Diagnostics**	LDT Design	Optical	Raman Spectroscopy	Fingertip, Skin	I
**GWave**	Hagar Tech	Electromagnetic	RF Spectroscopy	Wrist	C
**Movano Wearable CGM**	Movano	Electromagnetic	RF Spectroscopy	Wrist	C
**GlucoWear**	Afon Technology	Electromagnetic	RF/MW Spectroscopy	Wrist	C
**GlucoTrack**	Integrity Applications Ltd	Electromagnetic	Ultra-sound, application of electromagnetic waves and calorimetry	Earlobe	I
**Egm1000**	Evia Medical Technologies Limited		Ultra-sound, application of electromagnetic waves and thermal	Earlobe	I

C= continuous, I=Intermittent

*was Integrity Applications Ltd, ** heart rate, blood pressure, blood oxygen saturation, blood-flow velocity and ECG, *** CM=continuous monitor, SR= single read, LS =lifestyle, **** Optical Glucose Monitoring System, ***** D-SP= Sensor, pocket
